# Community-based digital mental health interventions for traumatic brain injury patients: A scoping review

**DOI:** 10.1371/journal.pmen.0000397

**Published:** 2025-08-19

**Authors:** Brandon G. Smith, Laura Hobbs, Tom Edmiston, Orla Mantle, Sara Venturini, Shobhana Nagraj, Charlotte J. Whiffin, Peter J. Hutchinson, Tom Bashford

**Affiliations:** 1 International Health Systems Group, Department of Engineering, University of Cambridge, Cambridge, United Kingdom; 2 NIHR Global Health Research Group on Acquired Brain and Spine Injury, University of Cambridge, Cambridge, United Kingdom; 3 Cambridge Public Health Interdisciplinary Research Centre, University of Cambridge, Cambridge, United Kingdom; 4 Department of Anaesthesia, East and North Hertfordshire NHS Trust, Stevenage, United Kingdom; 5 Division of Neurosurgery, Department of Clinical Neurosciences, University of Cambridge, Cambridge, United Kingdom; 6 Department of Public Health & Primary Care, University of Cambridge, Cambridge, United Kingdom; 7 College of Health, Psychology and Social Care, University of Derby, Derby, United Kingdom; 8 Division of Anaesthesia, University of Cambridge, Cambridge, United Kingdom; Instituto Federal do Maranhão: Instituto Federal de Educacao Ciencia e Tecnologia do Maranhão, BRAZIL

## Abstract

Traumatic brain injury (TBI) is a leading cause of long-term disability, often accompanied by mental health issues such as depression and anxiety. Digital mental health interventions (DMHIs) present promising opportunities for improving the management of these issues, offering solutions such as remote monitoring and outcome tracking through ecological momentary assessment. This scoping review aims to explore the current landscape of DMHIs and the use of patient-reported outcome measures (PROMs) in post-TBI populations. A systematic search across six databases identified 23 relevant studies, predominantly from high-income countries. Almost half of retrieved studies focused on mild TBI populations, with limited evidence reporting DMHI use exclusively in moderate or severe cases. The findings highlight the benefits of DMHIs, including real-time data collection, enhanced patient engagement, and the potential to improve care accessibility. However, challenges such as technology literacy, low response rates, and inconsistent measures of clinical efficacy were noted. Most interventions utilised asynchronous methods of communication, such as smartphone applications and SMS, with PROMs used to track emotional, behavioural, and psychological outcomes. A number of gaps were identified, including the need for more research in moderate and severe TBI cases, better integration into existing healthcare infrastructure, and standardisation of outcome measures. This review underscores the potential of DMHIs to enhance mental health care in TBI patients, while calling for more robust, user-centred designs and longer-term studies to ensure sustainability and effectiveness. Further, this review advocates for more interdisciplinary collaboration in the design and deployment of DMHIs, and the application of a systems-based approach to better integrate digital mental health technologies into TBI care pathways, with full consideration of people, systems, design, and risk. Future research should address these gaps to optimise post-injury care and outcomes for TBI patients when digital mental health solutions are implemented.

## Introduction

Traumatic brain injury (TBI) is a major cause of death and disability worldwide, with an estimated 69 million new cases occurring each year. [1] Generally, the severity of TBI (mild, moderate, severe) is determined at the time of injury through criteria such as the Glasgow Come Scale. [2] and can result in a wide range of physical, cognitive, emotional, and behavioural sequelae that can persist long-term and significantly impact quality of life. Mental health issues such as depression, anxiety, and post-traumatic stress disorder are commonplace following TBI, with prevalence rates much higher than in the general population. [3]

Monitoring mental health outcomes and overall recovery after TBI is crucial for optimising patient care and rehabilitation. Patient-reported outcome measures (PROMs) are increasingly recognised as valuable tools for assessing physical, mental, cognitive, social well being and functioning, and quality of life, from the patient’s perspective. Further, PROMs can provide temporally rich insights into the subjective experience of TBI that may not be captured by clinician-derived, objective clinical measures captured at infrequent scheduled timepoints alone. [4]

Digital mental health interventions (DMHIs) have potential to transform mental health care provision and access after TBI is substantial. [5,6] From text-messaging and feature phones through to smartphone- and web-based applications, these technologies offer opportunities for early detection, remote monitoring, outcome data collection, and personalised interventions accessible for patients and their carers in the community. Benefits of DMHIs extend to healthcare providers, enabling a richer, more comprehensive understanding of patient experience in the community, beyond the traditional bounds of a formal clinical setting. DMHIs may thereby facilitate a more personalised, responsive, precise, and patient-centered approach to post-TBI mental health. [4]

The unique physical, social, behavioural, and cognitive impairments associated with TBI and mental health comorbidities necessitate careful consideration in the design, implementation, and deployment of digital tools that seek to benefit such patient cohorts. Moreover, the complex healthcare ecosystems needed for these technologies to operate successfully demand a systematic approach to their development, integration, and continued use free of operational challenges.

The 2017 Engineering Better Care (EBC) framework, [7] developed by a partnership between the Royal Academy of Engineering, the Academy of Medical Sciences, and the Royal College of Physicians, offers a practical and holistic perspective for addressing challenges in healthcare improvement. It is structured around five key questions that guide improvement efforts:

1. Why are we doing this?
2. What is the problem?
3. Who should be involved?
4. What does ‘good’ look like?
5. What should we do next?

These questions are explored through four critical perspectives: ‘*People’*, ‘*System’*, ‘*Design’*, and ‘*Risk’*, which together form the foundation of the EBC’s systems approach. This approach emphasises understanding healthcare as a “system of systems,” where interdependent elements work together, with particular focus on the interactions between them. By viewing healthcare in this integrated manner, the EBC framework helps address the complexity of healthcare systems and ensures that improvement efforts are holistic, addressing technical, social, and human factors simultaneously.

By considering how digital mental health technologies for TBI align with these perspectives, we can gain crucial insights into their effectiveness, usability, and potential for successful implementation and sustainability of new services with respect to overcoming the contextual challenges that patients may face in their continued use.

Despite the growing interest in digital mental health interventions and PROMs for TBI, there remains a paucity in the literature depicting a comprehensive outline of the current landscape. We conducted a scoping review to map the existing literature, identify key concepts and gaps in knowledge, and inform future research and practice in this rapidly evolving field. By synthesising the current state of knowledge through the lens of the Engineering Better Care framework, this review aims to provide a nuanced understanding of digital mental health approaches and PROMs in TBI, guiding researchers, clinicians, and policymakers in advancing patient-centred care and outcomes for this population.

The objectives of this scoping review are to:

1. Identify and characterise digital mental health solutions and platforms used for patients with TBI;
2. Examine how PROMs are being collected and utilised in digital mental health approaches for TBI;
3. Explore the reported benefits, challenges, and limitations of digital approaches for mental health and PROMs in TBI populations;
4. Identify gaps in the current evidence base and areas for future research.

## Methods

This scoping review was guided by the comprehensive review framework by Arksey and O’Malley. [8] We included minor refinements suggested by Levac and colleagues [9] and the Joanna Briggs Institute (JBI). [10–12] This scoping review is reported in accordance with the Preferred Reporting Items for Systematic Reviews and Meta-Analyses extension for scoping reviews (PRISMA-ScR). [11]

This framework includes the following phases: (1) identifying the research question; (2) identifying relevant studies; (3) study selection; (4) charting the data; (5) collating, summarising, and reporting the results; and an optional (6) consultation exercise.

Unlike systematic reviews, scoping reviews do not require an antecedent protocol registration. [13] Notwithstanding, review objectives, eligibility criteria, and preliminary study characteristics to be extracted were determined a priori.

### Research question

*How have digital mental health solutions and patient-reported outcome measures for individuals with traumatic brain injury been designed and implemented, and which, if any, have considered elements of people, systems, design, and risk?*

In realising this overarching question, a number of sub-questions were posed, found in Table 1 below.

**Table 1:** Research sub-questions.

What types of digital mental health solutions and PROMs are being used for TBI populations, and in what settings? What are the reported benefits, challenges, and limitations of these digital approaches for mental health and PROMs in TBI populations? How have TBI survivors, carers, and healthcare providers been involved in the development and implementation of these technologies? What strategies have been employed to ensure user-centred design? How do these digital solutions and PROMs integrate with existing healthcare systems and workflows? What systemic factors have been considered in their implementation? What design principles and methodologies have been applied in developing these technologies for TBI populations? How have the specific cognitive and functional needs of TBI survivors been addressed in the design process? What potential risks (e.g., privacy concerns, exacerbation of symptoms, digital divide) have been identified in the use of these technologies for TBI populations, and how have these risks been mitigated? What gaps exist in the current evidence base regarding the design and implementation of digital mental health interventions and PROMs for TBI, particularly in relation to the Engineering Better Care framework?

### Eligibility criteria

The Population/Participants, Concept, and Context (PCC) framework [10,14] informed our eligibility criteria, search and data charting strategies. Eligibility criteria is outlined briefly in Table 2. A full PCC justification can be found in S1 Table.

**Table 2:** Eligibility (inclusion/ exclusion) criteria.

Inclusion criteria:
1) Any published original research with full texts available in English, including: primary studies, protocols, reports, editorials, opinion articles, letters, conference abstracts, theses, and book chapters; 2) Reports with a primary aim to describe, assess, or examine the use of digital health technologies to support the remote collection of patient outcome data, orientated to mental, affective, emotional, or psychological health; and 3) Paediatric and adult patient cohorts with a TBI of any severity, including mixed-pathology cohorts (e.g., TBI and post-traumatic stress disorder).
**Exclusion criteria:** 1) Secondary research (reviews); 2) Studies that report exclusively on outcomes related to family members or caregivers, without presenting any outcomes directly related to the target population of interest (e.g., patients, participants, or service users); or 3) No TBI population or involved healthy volunteers only.

### Search strategy

The following electronic databases were searched from database inception, with a final search executed on 15th September 2024 across databases: OVID Medline, OVID Embase, OVID Global Health, EBSCO PsycInfo, Scopus, and Web of Science. Additionally, we searched clinical trial registries (ClinicalTrials.gov), relevant conference proceedings, and scanned reference lists of included studies and relevant reviews to identify additional eligible studies. A grey literature search was conducted via Google Scholar using a combination of keywords aligned with our main concepts (‘head injury’, ‘brain injury’, ‘digital health’, ‘mental health’, ‘outcome’). The first 300 results (sorted by relevance) were screened to identify additional eligible studies, consistent with recommended practices for grey literature searching. and a number of head-injury, digital health, and mental health-focused journals (Journal of Neurotrauma, The Journal of Head Trauma Rehabilitation, Journal of Medical Internet Research, Journal of Telemedicine and Telecare, BMJ Mental Health, and BMC Psychiatry).

The search strategy was initially developed in an iterative manner for Ovid MEDLINE, and adapted for other databases. The final strategy was based on the review objective, combining concepts and terms related to “traumatic brain injury”, “mental health”, “digital health”, and “patient-reported outcomes”. The authors selected a number of ‘indicator papers’, of which are pre-identified articles that we expected to appear in the retrieved articles, to test the extensivity and quality of the strategy. A number of pilot searches were executed, comprising different variants of key terms and their combinations, prior to the development of a final comprehensive search strategy ready for cross-database translation. Search strategies for all selected databases are reported in S2 Table.

### Selection of sources of evidence

Following deduplication, two reviewers (BS, OM) independently screened titles and abstracts of all retrieved records against the eligibility criteria. Full texts of potentially relevant original research articles and protocols were then assessed independently by two reviewers (BS, OM). Any disagreements were resolved through discussion, or consultation with a third reviewer (TB). The study selection process was managed using the cloud-based Rayyan systematic review software. [15]

### Data charting

A standardised data charting form was developed in a cloud-based spreadsheet to extract relevant information from included studies. The form was piloted on a sample of 5 studies and refined iteratively. Two reviewers (BS, OM) independently extracted data from each included study, with any discrepancies resolved through discussion and involvement of a third reviewer (TB).

As per guidance for scoping reviews, no formal quality of evidence assessment or critical appraisal of the methodological quality of included studies was conducted. [13]

### Data items

The following data were extracted from each retrieved study:

- Study characteristics (e.g., author, year, country, study design);
- Population characteristics (e.g., sample size, TBI severity, time since injury);
- Digital mental health intervention implementation (e.g., technological medium or format, duration of intervention (e.g., sessions, intervals, duration);
- Clinician- and patient-reported outcome measures relevant to mental health utilised;
- Key findings related to the feasibility, acceptability, and effectiveness of the DMHI, including reported benefits, limitations, and challenges of digital approaches.

### Synthesis of results

The review results were organised thematically based on the data items charted. Quantitative data were summarised using descriptive statistics where appropriate.

In addition, all eligible articles were independently thematically coded by two reviewers (BS, OM) against the EBC framework domains of People, Systems, Design and Risk, and the three guiding questions associated with them. By consensus, the two reviewers assigned an arbitrary evaluation rating of N (not addressed), + (minimally addressed), ++ (adequately addressed), and +++ (thoroughly addressed) for each article with respect to the degree it addresses the EBC domains. This approach sought to offer a novel lens through which to view the evidence base, framed through a systems approach to health and care design and continuous improvement, providing an opportunity for the researchers to identify weaker EBC domains that needed to be addressed the future development and implementation of digital mental health solutions.

### Patient and Public Involvement (PPI)

During the development of this review, we conducted a Patient and Public Involvement (PPI) workshop, in collaboration with Cambridge Public Health and the Precision Health Initiative. The workshop engaged 18 participants from the local community, recruited through various outreach methods, and focused on gathering public input regarding digital health technologies, healthcare data, and AI in healthcare.

Through professionally facilitated discussions, participants provided valuable feedback highlighting the importance of tools to garner healthcare data in a community setting, and the burden of digital tools on users. The insights gathered directly influenced the design of our scoping review charting proforma, ensuring the inclusion of key aspects relevant to patient (and their proxies; family, carers) experiences. These included factors such as the which technological medium through which data was requested (e.g., SMS, smartphone application), whether proxies could interact with digital health technologies on behalf of patients, and the format in which the data was collected (e.g., if synchronous and involving real-time, 1:1 interaction with clinicians, or asynchronous data entry through an ‘offline’ platform). Additionally, considerations about the burden imposed by lengthy assessments (e.g., multiple PROMs or surveys in succession) versus their perceived value featured in discussions. This input from the public played a crucial role in ensuring that our efforts to retrieve and map the existing evidence base surrounding DMHIs was both patient-centric and sensitive to public concerns.

## Results

**Fig 1:**
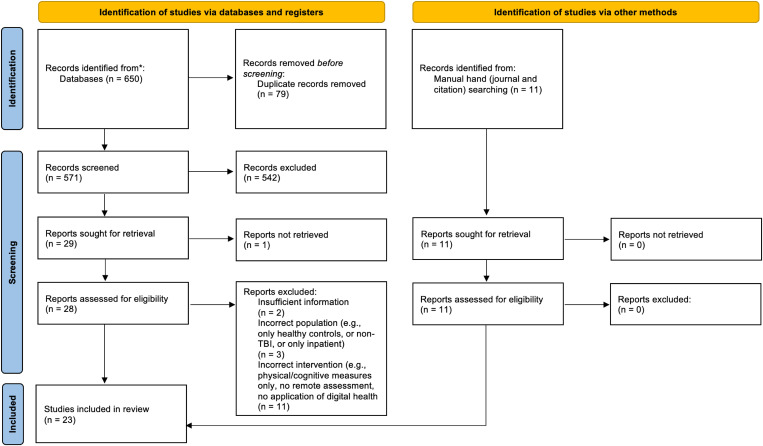
Preferred Reporting Items for Systematic Reviews and Meta-Analyses for Scoping Reviews (PRISMA-ScR) flowchart detailing the study selection process.

Executing the search strategy across the six selected electronic databases yielded a total of 650 potentially eligible citations. Following deduplication, 571 articles remained. A subsequent title and abstract screening yielded 40 citations for further full-text review. This final stage of screening concluded with 23 articles for inclusion, inclusive of 11 articles discovered through manual hand and citation searches of relevant journals and included articles, respectively. The charting proforma detailing reports for all articles is available in S3 Table. A full PRISMA-ScR flowchart depicting the study search, selection, and exclusion processes is depicted below in Fig 1. The PRISMA checklist is available in S1 Checklist.

### Study characteristics

Of the 23 articles retrieved, the majority were descriptive (n = 9) in design and included small prospective pilot feasibility studies of digital mental health interventions. [16–24]

The next most frequently adopted study design was quasi-experimental (n = 7) [25–31], and included prospective repeated measures [27,29,31] and non-randomised open-label clinical trials. [30] Observational studies were also prominent, with five articles reporting such designs, [32–36] and included prospective cohort pilot [33] and longitudinal observational studies. [32] The least prominent study design was experimental, with only two articles reporting its use, both of which comprising randomised controlled trials. [37,38]

The results of these studies were published as original primary articles (n = 21), with two publications of ‘Correspondence (Research Letter)’ [18] and ‘Conference Abstract’ [21] article types.

**Fig 2:**
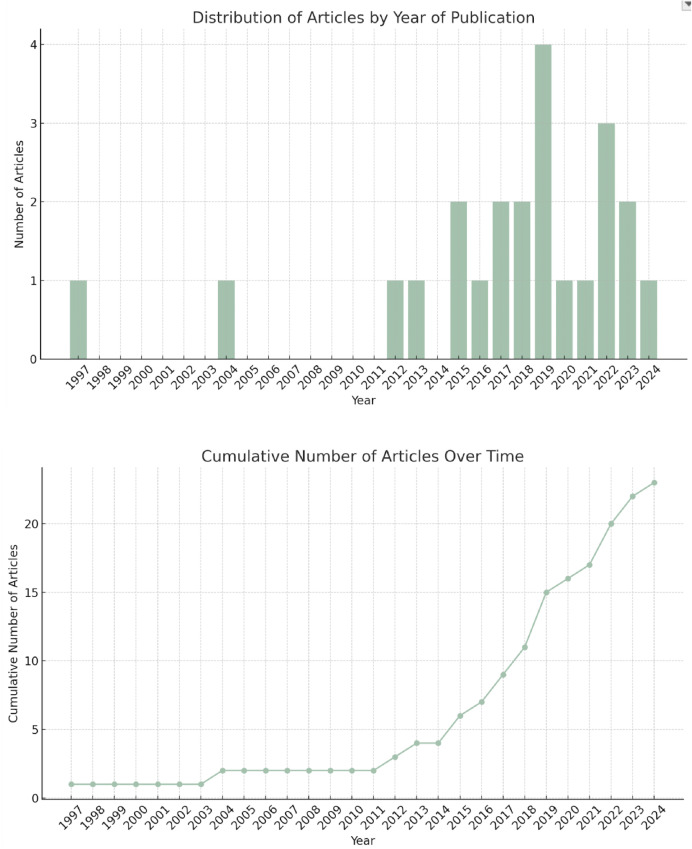
Distribution of articles by year of publication, and cumulative growth of the DMHI evidence base.

The majority of articles were published in the last decade, with one exception from 1997. [19] A visualisation of citations over time is presented below (Fig 2) as a distribution of articles by year of publication, and cumulative growth of the DMHI evidence base.

### International context

The adoption of digital mental health technologies is widespread across various countries. However, the vast majority of published studies (87%, n = 20) originated from high-income countries (HICs), with the USA contributing nearly 78% of the evidence base (n = 18) [16–20,24,26–31,33–38], followed by Canada (n = 1) [21] and The Netherlands (n = 1). [32] Low- and middle-income countries comprised the remaining 13% of the evidence base, with articles from Uganda (n = 2) [22,23] and India (n = 1) [39] respectively.

### Patient population and traumatic brain injury characteristics

Of the 23 included studies, 12 articles focused on exclusively adult populations (i.e., > 18 years of age), representing approximately 719 patients. Among these, the majority (n = 11) reported on civilian populations, [19–21,24–27,32,34,36,38] while 1 study specifically targeted veterans. [16]

Nine studies explored digital mental health interventions in paediatric populations (i.e., < 18 years of age), representing approximately 408 patients. Of these, the majority (n = 5) reported the use of digital mental health interventions within adolescent populations (i.e., 10 years of age and upwards). [28–31,37] One study reported a paediatric and adolescent population. [33] The remainder of these studies report general TBI patient cohorts. [17,18,23]

Two studies report paediatric-adult mixed populations (n = 2), one with an adolescent and young adult cohort [35], and another with an adult and paediatric cohort. [22]

The majority (n = 17, 74%) of articles report patient cohorts with TBI as their sole pathology. [18–21,23–31,33,35,37,38] The reports of remaining studies are of mixed pathology; two studies report of populations with TBI and a non-traumatic brain injury (i.e., acquired brain injury) [17,34], one study reports TBI and wider neuropathology (spina bifida, hydrocephalus, brain tumours) [22], one study reports TBI and head injury without TBI, amongst non-head injury trauma controls [36], one explicitly defines a TBI and stroke population [32], and one of TBI and PTSD. [16]

For all articles retrieved, with respect to TBI severity within the reported populations, the majority (48%) of DMHIs were implemented in cohorts of mild severity (n = 11), often referred to as ‘concussion’ or ‘mTBI’. [16,18,28–31,33,35–38] No studies exclusively reported DMHIs for Moderate or Severe severity patient cohorts. The second most reported cohort with respect to TBI severity was ‘all-severity’ cohorts (i.e., mild, moderate, and severe), comprising seven articles (30%). [19,22–27]

Where TBI severity was reported, the remaining (n = 2) articles report Mild-Moderate [21] and Moderate-Severe [20] patient cohorts. TBI severity was not reported in three (13%) studies. [17,32,34]

The majority (65%, n = 15) of articles report the use of DMHIs exclusively in a community setting (i.e., home, residential, or community treatment facility). One article reports the use of a DMHI in an outpatient clinic. [25] The remainder of articles report the introduction of DMHI at a period of care transition; four describe DMHI use starting in an emergency department (ED) before discharge to the community, [28,33,36,37] two describe their use at an outpatient (e.g., concussion) clinic and subsequently a community setting, [30,31] and one describes its use beginning in a neuroscience intensive care setting prior to transitioning to the community. [34]

### Time since injury at DMHI implementation

With respect to time since injury at the point of DMHI implementation, the retrieved articles are broadly grouped together by respective time frames of: less than 1 month, 1–3 months, 3–6 months, 6–12 months, and 12 months or more. Time since injury could be extracted in 83% (n = 19) of articles retrieved.

### Digital mental health intervention implementation and outcome measures utilised

Of all retrieved articles, four distinct communication modalities formed the basis of the DMHI: text-messaging (SMS) (n = 4, 17%), [33,35,36,38] including ‘text-messaging robots’(35), telephony (n = 7, 30%), [17,19–23,34] smartphone applications (n = 8, 35%), [18,24–26,29–31,37] including the use of gamified symptoms journals [30] and applications installed on Apple iPod Touch, [18] and custom devices (n = 1, 4%), including a custom touchscreen electronic device known as the ‘PsyMate’. [32] The remaining studies (n = 3, 13%) implemented DMHIs across more than one technology, including the use of SMS messages to direct the user to an online web survey platform (RedCap) [27], a gamified mobile learning environment available through an ‘mHealth app’ or website interface, [28] and the use of a personal digital assistant (PDA) for ecological momentary assessment, supplemented by longer-term SMS follow-up texts on the patient’s own handset. [16]

Over half of DMHIs were asynchronous in nature (n = 15, 65%), [16,18,24,26–33,35–38] and predominantly involved the use of smartphone application- and SMS/text-messaging-based solutions. All synchronous solutions (n = 8, 35%) [17,19–23,25,34], bar one, employed telephony as the DMH’s communication medium.

The majority of articles (n = 18, 78%) reported no use of proxy (i.e., a family member, carer, or other professional assisting in patient assessment) within the DMHI. [16–19,21,24,26–33,35–38] Three articles (9%) report the partial use of a proxy, either to provide additional information at each contact, [20,34] or to assist in initiating contact with the patient. [22] Two further article describe wholly interacting with proxies in DMHI implementation; one instance describes the assessment of GOSE-Peds and general quality of life survey through a parent of a paediatric TBI patient, [23] and another describes a clinician-derived GOSE score of adult patients in an outpatient clinical setting. [25]

With respect to the number of assessment sessions and their frequency, four styles of delivery emerged: a) one assessment (n = 6, 26%), [17,21–23,25,34] b) once daily over a prescribed period (e.g., until symptom resolution, or a set time period) (n = 6, 26%) [16,24,27,28,30,37] c) multiple times daily over a prescribed period (n = 8, 35%) [18,26,29,31,32,35,36,38] and d) multiple times, not daily, over a prescribed period (n = 3, 13%). [19,20,33]

A set of 27 outcome measures were identified in all articles retrieved (Table 3). Multiple studies (n = 8) employed two or more established outcome measures in their DMHI implementations, such as in the form of a battery, [17,22–24,26,33,35,36] whilst a few studies relied on internally designed measures, scores, and scales for the remote evaluation of mental health status for TBI patients. [16,20,22,23]

**Table 3:** Outcome measures utilised in all retrieved articles.

Outcome measure (and derivatives)	Abbreviation	Citations/ Number of implementations
Neurobehavioral Rating Scale	NRS	[19]
Rivermead Post-concussion Questionnaire Brief Derivative	RPQ N/A	[21,36] [38]
Patient Health Questionnaire-2 Patient Health Questionnaire-9	PHQ-2 PHQ-9	[24] [26]
General Anxiety Disorder-2 General Anxiety Disorder-7	GAD-2 GAD-7	[24] [26]
Positive and Negative Affect Schedule	PANAS	[24,26,32]
Post-concussion Symptom Score	PCSS	[18,28,29,31,35]
Post-concussion Symptom Inventory	PCSI	[33,37]
Pain Catastrophizing Scale	PCS	[33]
Concussion Symptom Severity Score	CSSS	[35]
Sport Concussion Assessment Tool-2	SCAT-2	[35]
Sport Concussion Assessment Tool-3	SCAT-3	[30]
EuroQol 5-dimension, 5-level	EQ-5D-5L	[34]
Glasgow Outcome Scale Extended Paediatric derivative Kannada language	GOS-E GOS-E-PEDS N/A	[17,22,23] [25]
PTSD Checklist Civilian	PCL	[36]
PedsQL Family Impact Module	PedsQL PedsQL-FIM	[17]
Behavioral Assessment Screening Tool mHealth short derivative	BAST BAST_mHealth_	[27]
Custom 32-item PROM derived from Symptom Checklist-6, BriefCOPE, Beck Depression Inventory II, and general 5-point well being question	N/A	[16]
Non-specific, structured review of 17 domains, including behavioural concerns, emotional function	N/A	[20]
General survey items pertaining to quality of life, school and work function, psychosocial function	N/A	[22]
Parent-oriented quality of life interview including psychosocial function of child with brain injury (friendly, responsible, displays emotion, behaves with parents)	N/A	[23]

The most frequently used measures, including derivatives, by citation were the global Post-concussion Symptom Score (PCSS) (n = 5) [18,28,29,31,35] and the Glasgow Outcome Scale Extended (GOS-E) (n = 4) [17,22,23,25], followed by the Rivermead Post-concussion Questionnaire (RPQ) (n = 3) [21,36,38] and PANAS (n = 3)

### Thematic coding of retrieved articles against the engineering better care framework

**Fig 3:**
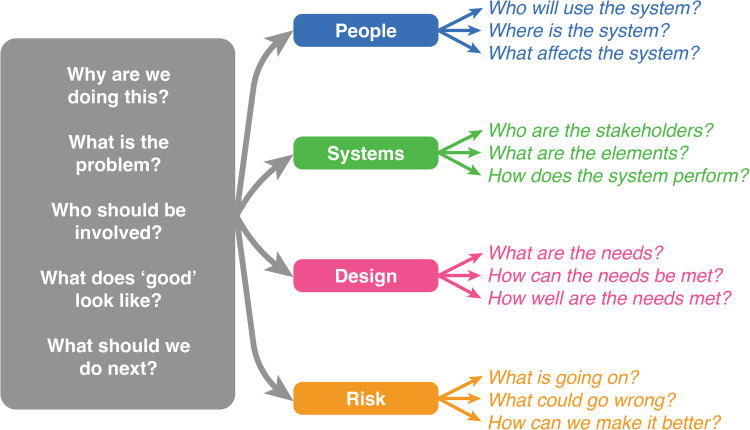
The ‘systems approach’ framework to health and care design and continuous improvement, adapted from *Engineering Better Care.* [7].

Each retrieved article was assigned a rating based on the extent to which it addressed the domains of the EBC framework (Fig 3), with the evaluation categories ranging from N (not addressed) to +++ (thoroughly addressed).

The evaluation of each article against the four domains is summarised in Table 4; an aggregate score has been calculated for each domain and its respective subdomains (i.e., N = 0, + = 1, ++ = 2, +++ = 3).

**Table 4:** Retrieved articles coded against the Engineering Better Care framework, with individual subdomain scores represented in parentheses.7).

Citation	People *Who will use the system?* *Where is the system?* *What effects the system?*	Systems *Who are the stakeholders?* *What are the elements?* *How does the system perform?*	*Design* *What are the needs?* *How can the needs be met?* *How well are the needs met?*	Risk *What is going on?* *What could go wrong?* *How can we make it better?*
***Dombovy, et al. (1997)***	**4** (++/ + / +)	**5** (++/ + / ++)	**5** (++/ + / ++)	**3** (+/ + / +)
***Bell, et al. (2004)***	**6** (++/ ++/ ++)	**6** (++/ ++/ ++)	**5** (++/ ++/ +)	**4** (++/ + / +)
***Smith, et al. (2012)***	**7** (+++/ ++/ ++)	**6** (++/ ++/ ++)	**5** (++/ ++/ +)	**4** (++/ + / +)
***Suffoletto, et al. (2013)***	**4** (++/ + / +)	**7** (++/ ++/ +++)	**7** (++/ ++/ +++)	**4** (++/ + / +)
***Juengst, et al. (2015)***	**7** (+++/ ++/ ++)	**8** (+++/ +++/ ++)	**8** (+++/ +++/ ++)	**9** (+++/ +++/ +++)
***Anthony, et al. (2015)***	**4** (++/ + / +)	**7** (++/ ++/ +++)	**6** (++/ + / +++)	**5** (++/ + / ++)
***Wiebe, et al. (2016)***	**5** (++/ ++/ +)	**4** (+/ + / ++)	**3** (+/ + / +)	**3** (+/ + / +)
***Worthen-Chaudhari, et al. (2017)***	**6** (++/ ++/ ++)	**7** (++/ ++/ +++)	**8** (++/ +++/ +++)	**4** (++/ + / +)
***Thibault-Halman, et al. (2017)***	**3** (+/ + / +)	**3** (+/ + / +)	**3** (+/ + / +)	**1** (+/ N/ N)
***Xu, et al. (2018)***	**7** (+++/ ++/ ++)	**6** (++/ ++/ ++)	**6** (++/ ++/ ++)	**4** (++/ + / +)
***Pacella, et al. (2018)***	**5** (++/ + / ++)	**6** (++/ ++/ ++)	**6** (++/ + / +++)	**3** (+/ + / +)
***Lenaert, et al. (2019)***	**6** (+++/ ++/ +)	**7** (++/ ++/ +++)	**7** (++/ ++/ +++)	**4** (++/ + / +)
***Sufrinko, et al. (2019)***	**6** (+++/ ++/ +)	**7** (++/ ++/ +++)	**7** (++/ ++/ +++)	**3** (+/ + / +)
***Juengst, et al. (2019)***	**6** (+++/ ++/ +)	**6** (++/ ++/ ++)	**7** (++/ ++/ +++)	**4** (++/ + / +)
***Vaca, et al. (2019)***	**8** (+++/ +++/ ++)	**6** (++/ ++/ ++)	**8** (+++/ ++/ +++)	**4** (++/ + / +)
***Schlichter, et al. (2020)***	**6** (+++/ ++/ +)	**7** (++/ ++/ +++)	**8** (+++/ ++/ +++)	**3** (+/ + / +)
***Trbovich, et al. (2021)***	**4** (++/ + / +)	**6** (++/ ++/ ++)	**5** (++/ + / ++)	**3** (+/ + / +)
***Nabasny, et al. (2022)***	**7** (+++/ ++/ ++)	**7** (++/ +++/ ++)	**6** (++/ ++/ ++)	**4** (++/ + / +)
***Schmidt, et al. (2022)***	**4** (++/ + / +)	**6** (++/ ++/ ++)	**6** (++/ ++/ ++)	**4** (+/ + / ++)
***Schoenfeld, et al. (2022)***	**6** (+++/ ++/ +)	**7** (++/ ++/ +++)	**7** (++/ ++/ +++)	**3** (+/ + / +)
***Shukla, et al. (2023)***	**4** (++/ + / +)	**7** (++/ ++/ +++)	**6** (+/ ++/ +++)	**3** (+/ + / +)
***Wade, et al. (2023)***	**6** (+++/ ++/ +)	**6** (++/ ++/ ++)	**7** (++/ ++/ +++)	**4** (++/ + / +)
***Riggall, et al. (2024)***	**5** (++/ ++/ +)	**5** (++/ + / ++)	**6** (++/ ++/ ++)	**4** (++/ + / +)
***Total domain scores***	**People: 116**	**Systems: 131**	**Design: 132**	**Risk: 78**

Note: Individual subdomain scores are represented in parentheses.

As shown in Table 4, the majority of articles adequately addressed the domains of Systems and Design, with scores of 131 (63%) and 132 (64%) respectively against a possible 207 (100%). However, the domains of People and Risk were less consistently addressed (scores of 56% and 38% respectively), particularly with regards to addressing subdomains of People: “*Where is the System?*” and “*What affects the system?*”, and across all subdomains of Risk: “*What is going on?*”, “*What could go wrong?*”, and “*How could it be better?*” suggesting these areas are either underexplored or underreported within the existing evidence base for post-TBI digital mental health.

## Discussion

In the context of this scoping review, which focusses on digital mental health solutions for individuals following traumatic brain injury (TBI) with an emphasis on patient-reported outcome measures (PROMs) and data collection via technology, significant insights emerge from the aims of the reviewed studies. The articles collectively highlight a potential role of digital tools, including mobile health (mHealth) applications, text messaging, and telephone follow-up, in advancing the assessment and management of mental health and functional outcomes in TBI populations.

A common theme across many studies is the feasibility and effectiveness of collecting PROMs through technology. Whether assessing neuropsychological, emotional, or social outcomes, the studies consistently seek to determine whether digital platforms can accurately and efficiently gather data on patients’ symptoms and recovery trajectory. This reflects the broader shift toward leveraging mHealth technologies to extend the reach of clinical assessment beyond traditional in-person visits, making continuous monitoring and long-term follow-up more accessible, particularly in populations with barriers to frequent clinical engagement.

Furthermore, a number of studies focus on the use of real-time data collection through ecological momentary assessment (EMA) and mobile ecological momentary assessment (mEMA), which allows for the capture of symptom fluctuations in patients’ everyday environments. This approach not only provides a more dynamic understanding of patients’ mental health states but also enhances the accuracy of symptom tracking, compared to retrospective reporting methods. This ability to track within-day or daily symptom variation—such as mood, fatigue, and cognitive symptoms—provides valuable data that can inform timely interventions and more personalised care plans.

Additionally, whilst beyond the scope of this review, several studies investigate the potential for mHealth solutions to deliver behavioural and psychoeducational support directly to patients. These solutions range from text messaging-based educational programs to mobile apps designed to promote self-management and recovery. Such interventions, aimed at reducing the severity of post-concussive symptoms or motivating adherence to rehabilitation programs, show promise not only for symptom management but also for patient engagement and empowerment in the recovery process.

The review also reveals a growing interest in assessing the usability and scalability of these digital mental health solutions in varied populations, including youth, veterans, and individuals in low-resource settings. The ability to conduct long-term follow-ups, particularly through culturally and linguistically adapted phone surveys or mobile applications, highlights the versatility of these tools in addressing the diverse needs of TBI patients globally.

Many articles excluded at the title and abstract stages of review concerned the application of digital health (e.g., telephone) to gather pre- and post-intervention outcome measures (e.g., cognitive behavioural therapy via telerehabilitation), rather than the collection of such outcome data forming the basis of the article itself.

Our review reveals several key benefits of digital mental health approaches for TBI populations. First, these technologies allow for real-time, high-frequency data collection in naturalistic settings, enhancing the granularity and ecological validity of symptom tracking. For example, a number of studies demonstrated the feasibility of using SMS- and smartphone application-based EMA to capture within-day symptom variation, offering a more accurate picture of fluctuating post-concussive symptoms, inclusive of mental health and wellbeing, than traditional, less frequent assessments. Furthermore, mHealth tools, such as apps with social gaming elements, were shown to improve patient engagement, particularly in younger populations, facilitating long-term self-management and reporting of symptoms. Digital platforms also reduced geographical and logistical barriers to care, as evidenced by telephone follow-up programs in Uganda that effectively reached underserved populations and identified unmet rehabilitation needs.

However, important challenges were also identified. One key issue surrounding engagement and compliance was technology literacy, as several studies perceived that participants with lower digital familiarity or severe cognitive impairments struggled to use mHealth tools effectively.

Additionally, technical difficulties, such as difficulties with prompt notification systems (e.g., devices blocking notifications), were cited as barriers to reliable data collection. Low response rates in certain studies, particularly as time since injury increased, further highlight the challenge of maintaining patient engagement over long-term follow-up.

The efficacy of digital mental health interventions—commonly assessed through metrics such as compliance, attrition, or response rates—was inconsistently reported across the studies reviewed. This lack of standardised reporting complicates the systematic comparison of different technological approaches and hinders the assessment of outcomes across diverse populations with varying levels of severity and demographic characteristics. While most studies aimed to evaluate the effectiveness of their technologies by analysing metrics such as response rates relative to the number of prompts sent or the number of patients reached after a specified number of contact attempts, more research and initiatives are needed to address this inconsistency. For instance, one study reported a 61% response rate after five contact attempts, [23] whereas another reported a 94% response rate by the second attempt. [34]

Lastly, the integration of PROMs into digital mental health solutions shows considerable promise for enhancing the efficiency and reach of outcome assessments in TBI care. Studies consistently demonstrated that digital tools, such as mobile apps and SMS systems, could send and retrieve PROM data at higher frequencies and in real-world environments, offering a more dynamic understanding of patients’ recovery trajectories. For instance, EMA and mHealth interventions provided valuable insights into day-to-day symptom variability, enabling clinicians to track changes in real time and potentially intervene more quickly when needed. This aligns with broader trends in patient-centred outcome measurement in rehabilitation research, where timely and continuous data collection is increasingly recognised as essential for personalised care. [40]

## Evidence gaps

This review has identified several important gaps in the current evidence base:

### Lack of co-design and stakeholder engagement, and need for a systems approach to DMHI design and implementation

Firstly, a substantive gap identified in the review is the insufficient application of a *systems approach,* a priori, in the design, implementation, and evaluation of digital mental health interventions for traumatic brain injury (TBI). While the majority of articles map to a handful of EBC domains, few, if any, report the use of a pragmatic, systems-led approach to their DMHI. This includes limited co-design with stakeholders, poor integration into existing healthcare workflows, inadequate user-centred design tailored to cognitive impairments, weak risk management, and a lack of iterative feedback loops for continuous improvement. Addressing this gap is crucial for developing effective, sustainable, and user-friendly digital health solutions that can seamlessly integrate into complex healthcare systems and meet the evolving needs of TBI patients.

### Limited research on long-term outcomes and sustainability of DMHI beyond the proposed research

Many studies focus on short-term outcomes of DMHI implementation (up to 90 days), but there is a noticeable gap in evidence regarding the long-term efficacy of digital mental health solutions for managing TBI-related mental health issues. There is a need for studies that detail DMHI implementation outside of a prescribed research environment, that assess long-term patient engagement, sustained mental health improvements, and functional outcomes beyond this short period to fully understand the impact of these interventions over time and their sustainability when translated wholly into a clinical environment. Long-term recovery from TBI often involves fluctuating mental health and functional abilities, and a multi-disciplinary approach to treatment and rehabilitation, making sustained monitoring critical.

### Under-representation of moderate and severe TBI populations

Although all-severity populations comprised almost a third of articles, most of the studies included a focus on patients with mild TBI. No studies exclusively reported the application of DMHIs in Moderate or Severe TBI populations. This leaves a significant gap in understanding how digital mental health interventions and PROMs might be adapted for and used by individuals with more severe mental, cognitive, and physical impairments, or how family members or carers may be suitably integrated as proxies reporting on behalf of the patient. More severe TBIs often result in more complex sequelae and therefore needs, including greater social, cognitive, and functional challenges. Digital tools must be tailored to meet these needs, but the lack of research in this area limits the development of appropriate interventions.

### Integration of DMHI within existing systems and digital health infrastructure

Of the studies retrieved, there is very limited evidence on how digital mental health interventions and PROMs are integrated into existing healthcare workflows. Studies rarely explore how these technologies can be seamlessly adopted into routine clinical care or how they interact with other health services, both in a clinical and community setting, which is crucial for their sustainability and scalability. Arguably, for DMHIs to be sustainable in the longer-term, they must be effectively integrated into existing healthcare infrastructure, such as electronic health records and health information systems. A lack of research in this area presents a barrier to scaling these interventions and ensuring they provide continuous, real-time support of TBI patients as part of a comprehensive care plan.

### Lack of outcome measure standardisation and inconsistent measures of efficacy

While many studies demonstrate the feasibility of using digital mental health technologies, fewer studies provide robust evidence of their longitudinal clinical efficacy. Some 27 outcome measures were identified to have been utilised in post-TBI DMHI studies to date. Though a number of measures were used more frequently, such as the PCSS, PANAS, and GOS-E, there is a notable lack of standardised outcome measures or batteries that allow for consistent evaluation across studies, making it difficult to compare the effectiveness of different DMHI interventions or draw meaningful conclusions about their impact on mental health outcomes. Further, the inconsistent reporting of key outcome measures of DMHI efficacy (e.g., symptom reduction, mental health improvements) additionally hampers the ability to assess the true effectiveness of digital mental health tools, highlighting the additional need for standardised outcome metrics following DMHI implementation across studies.

### Paucity of research exploring DMHIs in low-resource settings

Despite the increasing adoption of digital mental health interventions (DMHIs) globally, there is a marked disparity in the geographical distribution of research, with the vast majority of published studies originating from HICs. Notably, the majority of the HIC-originating evidence base stems from the USA alone, with smaller contributions from other HICs such as Canada and the Netherlands. In contrast, LMIC-based research accounted for only 13% of the evidence base, with a small number of articles emerging from Uganda and India. Strikingly, LMICs experience almost three times as many cases of TBI than LMICs. [1]

This significant imbalance in research representation highlights a critical gap in understanding the feasibility, acceptability, and effectiveness of DMHIs for individuals in resource-constrained settings where TBI remains a pressing public health challenge.

The limited exploration of DMHIs in LMICs presents several challenges and knowledge gaps. Firstly, technological and infrastructural constraints may hinder the successful implementation of these interventions, particularly in regions with low smartphone penetration, unreliable internet access, or limited access to digital literacy training. Existing DMHIs, largely designed and tested in HICs, may not be directly transferable to LMIC contexts without adaptation to address these barriers. [4]

Secondly, healthcare system integration and scalability remain unexplored in settings where mental health services are already under-resourced. The effectiveness of DMHIs in HICs often relies on well-established healthcare infrastructure and specialist support, raising concerns about their adaptability in regions with shortages of mental health professionals and limited healthcare funding.

Furthermore, cultural and contextual differences play a crucial role in shaping mental health perceptions and help-seeking behaviors, [41] yet there is a lack of research exploring how DMHIs can be tailored to diverse cultural contexts in LMICs. Affordability and accessibility may pose significant challenges, as the cost of digital interventions, including mobile data costs and device availability, may be prohibitive for many individuals in low-resource settings. [42] Without an emphasis on cost-effectiveness and equitable access, the potential of DMHIs to bridge the mental health treatment gap in LMICs remains limited.

Addressing this disparity requires a concerted effort to expand research collaborations between HICs and LMICs, ensuring that DMHIs are tested in diverse settings with meaningful stakeholder engagement. Future studies should explore alternative digital solutions, such as SMS- or community-supported interventions, that do not rely on high-speed internet connectivity. Moreover, research should investigate how caregivers, community health workers, and non-specialist providers can be integrated into DMHI delivery models to enhance accessibility in low-resource settings.

By broadening the evidence base to include LMICs, DMHIs can be adapted to meet the needs of a wider, more diverse population, ensuring that digital mental health solutions are equitable and scalable across global healthcare landscapes.

### Justification for a centralised, transdisciplinary register of outcome measures

Lastly, in lieu of the above evidence gaps, herein we provide justification for a centralised, transdisciplinary register of outcome measures.

There is a growing number of outcome measures available for traumatic brain injury, both patient and clinician reported, with estimates of over 1,000 available to select. [43] Outcome measures are often grouped according to the International Classification of functioning, Disability and Health (ICF) framework domains of Body Function, Activities and Participation and Body Structure, [44] though measures often span multiple ICF domains owing to the content they comprise. [39,45]

In 2016, the TBI EDGE Task Force evaluated and mapped some 88 outcome measures against the ICF, culminating in a 4-point outcome measure rating scale indicating psychometric properties (validity and reliability) and clinical utility (ease of administration, measure licensing). Such measures were also pooled with respect to their recommended context of deployment (e.g., acute setting, outpatient setting, research, patient dependence of ambulation). [45]

However, as found in conducting this review, we argue that the sheer number of outcome measures available poses a challenge for those wishing to select and apply an appropriate outcome measure. It is widely accepted that no single outcome measure can encompass the biopsychosocial and wider sequelae that TBI imposes on an individual or their families and carers; [39] in practice, it is often necessary to deliver multiple PROMs in an assessment battery format to garner a suitable insight, compounding the challenges of selecting an appropriate array of measures with the additional challenge of not overselecting in lieu of the additional patient and physician burden imposed in their use, which in turn, may have deleterious effects on the quality and validity of the data retrieved. [46,47]

In this respect, we propose the development of a novel health outcome measure register detailing both retrospective and prospective instrument validation studies, serving as a living review of measures and providing an itemised, evidence-based account of a measure’s clinical or academic utility, comprising an audit trail of development and validation studies, the clinical pathologies each measure is relevant for, which biopsychosocial or ICF domain it purports to cover, licensing terms (as appropriate), and cross-cultural adaptations (e.g., language) constructed for each measure, in turn empowering users of outcome measures in ensuring that their selection is contextually appropriate, and may lead to a modified core outcome set for TBI that centers on patient-reported outcome measures [48–51].

## Strengths

To our knowledge, this review is among the first to map the current global evidence base and garner a comprehensive understanding of how digital mental health interventions and patient-reported outcome measures are implemented in patient cohorts of whom have sustained a traumatic brain injury.

One of the particular strengths of this scoping review is the adoption of thematic coding to map the existing literature against the established Engineering Better Care framework for healthcare improvement. This process fostered an evaluation of the literature through a systems-based lens, and facilitated a holistic perspective that showcases the consideration of people, systems, design, and risk within the current evidence base.

Another strength lies in the decision to conduct a scoping review rather than a systematic or meta-analytic review. Given the rapidly evolving nature of DMHIs for TBI, a scoping review allows for the mapping of this emerging field, identifying key concepts, gaps, and future research directions without limiting the review to only high-quality trials or excluding early-phase feasibility studies. This method provides a broad overview of the literature and accommodates the inclusion of diverse study types, including pilot studies, observational data, and qualitative research, which are particularly important when investigating a novel and evolving topic like digital interventions for TBI. Scoping reviews are also useful for informing future research priorities, making them an ideal approach for topics with emerging but fragmented evidence.

## Limitations

Despite the strengths of the scoping review, it does present some limitations. First is the lack of formal quality appraisal for the included studies, a limitation inherent to the scoping review methodology. While each article was thematically coded with respect to the EBC framework, a future systematic review may benefit from appraising each article and its respective study design.

Furthermore, the inclusion of only English-language articles may introduce language bias, potentially excluding relevant studies from non-English-speaking regions, which are especially important when considering the global reach of digital health technologies. Lastly, while scoping reviews are beneficial for mapping broad areas of research, they are not designed to generate definitive conclusions about the effectiveness of interventions. Therefore, this review cannot firmly address the clinical impact or long-term outcomes of the DMHIs evaluated, particularly as many of the included studies are unpowered, small-scale pilot or feasibility studies with short follow-up periods. Further research, experimental in nature (randomised controlled trials), is warranted to confirm the effectiveness of these interventions in improving mental health outcomes post-TBI.

## Conclusions

This scoping review provides a comprehensive map of the current landscape of DMHIs for individuals with all-severity traumatic brain injury. Our findings highlight the potential of digital approaches to enhance community-based mental health care and outcomes assessment in this population, while also identifying important challenges and areas for future research.

In conclusion, digital mental health solutions hold significant potential for improving community-based data collection and patient support following all-severity TBI. However, further efforts are required to ensure these solutions are risk-mitigated, patient-centred, and sustainable for long-term use, while delivering net benefits to all stakeholders. An interdisciplinary systems approach provides a promising and practical framework for the development, implementation, and long-term use of these solutions.

## Supporting information

S1 Table Full justification of PCC criteria employed in article screening. (S1_Table.DOCX)

S2 Table Search strategies across selected databases. (S2_Table.DOCX)

S3 Table Charting proforma of all articles retrieved (n = 23). (S3_Table.DOCX)

S1 Checklist Preferred Reporting Items for Systematic reviews and Meta-Analyses extension for Scoping Reviews (PRISMA-ScR) Checklist. (S1_Checklist.PDF)

## References

[1] Dewan MC, Rattani A, Gupta S, Baticulon RE, Hung YC, Punchak M, et al. Estimating the global incidence of traumatic brain injury. J Neurosurg. 2018;1–18. 10.3171/2017.10.JNS17352 29701556

[2] Teasdale G, Jennett B. Assessment of coma and impaired consciousness. A practical scale. Lancet. 1974;2(7872):81–4. doi: 10.1016/s0140-6736(74)91639-0 4136544

[3] Schwarzbold M, Diaz A, Martins ET, Rufino A, Amante LN, Thais ME, et al. Psychiatric disorders and traumatic brain injury. Neuropsychiatr Dis Treat. 2008;4(4):797–816. doi: 10.2147/ndt.s2653 19043523 PMC2536546

[4] Smith BG, Tumpa S, Mantle O, Whiffin CJ, Mee H, Solla DJF, et al. Remote Follow-Up Technologies in Traumatic Brain Injury: A Scoping Review. J Neurotrauma. 2022;39(19–20):1289–317. doi: 10.1089/neu.2022.0138 35730115 PMC9529313

[5] Avramovic P, Rietdijk R, Attard M, Kenny B, Power E, Togher L. Cognitive and Behavioral Digital Health Interventions for People with Traumatic Brain Injury and Their Caregivers: A Systematic Review. J Neurotrauma. 2023;40(3–4):159–94. doi: 10.1089/neu.2021.0473 35819294

[6] Welsh ET, McIntosh JE, Vuong A, Cloud ZCG, Hartley E, Boyd JH. Design of digital mental health platforms for family member cocompletion: scoping review. J Med Internet Res. 2024;26:e49431. 10.2196/49431 PMC11255536 38959030

[7] Royal Academy of Engineering. Engineering Better Care: A Systems Approach to Health and Care Design and Continuous Improvement. 2017. https://raeng.org.uk/

[8] Arksey H, O’Malley L. Scoping studies: towards a methodological framework. Int J Soc Res Methodol. 2005;8(1):19–32.

[9] Levac D, Colquhoun H, O’Brien KK. Scoping studies: advancing the methodology. Implement Sci. 2010;5:69. doi: 10.1186/1748-5908-5-69 20854677 PMC2954944

[10] Aromataris E, Munn Z. JBI Reviewer’s Manual. JBI. 2019.

[11] Tricco AC, Lillie E, Zarin W, O’Brien KK, Colquhoun H, Levac D, et al. PRISMA Extension for Scoping Reviews (PRISMA-ScR): Checklist and Explanation. Annals of Internal Medicine. 2018;169(7):467–73. 30178033 10.7326/M18-0850

[12] Moher D, Liberati A, Tetzlaff J, Altman DG, PRISMA Group. Preferred reporting items for systematic reviews and meta-analyses: the PRISMA statement. BMJ. 2009;339(jul21 1):b2535. 10.1136/bmj.b2535 PMC2714657 19622551

[13] Munn Z, Peters MDJ, Stern C, Tufanaru C, McArthur A, Aromataris E. Systematic review or scoping review? Guidance for authors when choosing between a systematic or scoping review approach. BMC Med Res Methodol. 2018;18(1):143. doi: 10.1186/s12874-018-0611-x 30453902 PMC6245623

[14] Aromataris E, Munn Z. JBI manual for evidence synthesis. JBI. 2020.

[15] Ouzzani M, Hammady H, Fedorowicz Z, Elmagarmid A. Rayyan-a web and mobile app for systematic reviews. Syst Rev. 2016;5(1):210. doi: 10.1186/s13643-016-0384-4 27919275 PMC5139140

[16] Smith B, Harms WD, Burres S, Korda H, Rosen H, Davis J. Enhancing behavioral health treatment and crisis management through mobile ecological momentary assessment and SMS messaging. Health Informatics J. 2012;18(4):294–308. doi: 10.1177/1460458212445349 23257059

[17] Riggall EA, Slomine BS, Suskauer SJ, Borda A, Lahey S, Ludwig NN. Caregiver and family functioning after pediatric disorder of consciousness: telephone-based outcome assessment. Brain Inj. 2024;38(2):99–107. doi: 10.1080/02699052.2024.2304884 38328910

[18] Wiebe DJ, Nance ML, Houseknecht E, Grady MF, Otto N, Sandsmark DK, et al. Ecologic Momentary Assessment to Accomplish Real-Time Capture of Symptom Progression and the Physical and Cognitive Activities of Patients Daily Following Concussion. JAMA Pediatr. 2016;170(11):1108–10. doi: 10.1001/jamapediatrics.2016.1979 27617669

[19] Dombovy ML, Olek AC. Recovery and rehabilitation following traumatic brain injury. Brain Inj. 1997;11(5):305–18. doi: 10.1080/026990597123467 9146836

[20] Bell KR, Hoffman JM, Doctor JN, Powell JM, Esselman P, Bombardier C, et al. Development of a telephone follow-up program for individuals following traumatic brain injury. J Head Trauma Rehabil. 2004;19(6):502–12. doi: 10.1097/00001199-200411000-00007 15602312

[21] Thibault-Halman G, Fenerty L, Taylor P, Kureshi N, Walling S, Clarke DB. P.016 Early telephone follow-up for traumatic brain injury patients using the Rivermead post-concussion symptoms questionnaire. Can J Neurol Sci. 2017;44(S2):S17-8.

[22] Xu LW, Vaca SD, Nalwanga J, Muhumuza C, Vail D, Lerman BJ, et al. Life after the neurosurgical ward in sub-Saharan Africa: Neurosurgical treatment and outpatient outcomes in Uganda. World Neurosurg. 2018;e153-60. 10.1016/j.wneu.2018.01.204 29427813

[23] Vaca SD, Xu LW, Nalwanga J, Muhumuza C, Lerman BJ, Kiryabwire J, et al. Long-term follow-up of pediatric head trauma patients treated at Mulago National Referral Hospital in Uganda. J Neurosurg Pediatr. 2018;23(1):125–32. 30485178 10.3171/2018.7.PEDS17601

[24] Juengst SB, Terhorst L, Kew CL, Wagner AK. Variability in daily self-reported emotional symptoms and fatigue measured over eight weeks in community dwelling individuals with traumatic brain injury. Brain Inj. 2019;33(5):567–73. doi: 10.1080/02699052.2019.1584333 30836017

[25] Shukla D, Thombre BD, Baby P, Palaninathan J, Subramanian S, Prathyusha PV, et al. Validity of Glasgow outcome scale-extended (GOSE) mobile application for assessment of outcome in traumatic brain injury patients. Brain Injury. 2023;37(10):1215–9. 37269250 10.1080/02699052.2023.2218649

[26] Juengst SB, Graham KM, Pulantara IW, McCue M, Whyte EM, Dicianno BE, et al. Pilot feasibility of an mHealth system for conducting ecological momentary assessment of mood-related symptoms following traumatic brain injury. Brain Inj. 2015;29(11):1351–61. doi: 10.3109/02699052.2015.1045031 26287756

[27] Nabasny A, Rabinowitz A, Wright B, Wang J, Preminger S, Terhorst L, et al. Neurobehavioral symptoms and heart rate variability: feasibility of remote collection using mobile health technology. J Head Trauma Rehabil. 2022;37(3):178–88. 35125433 10.1097/HTR.0000000000000764 PMC9203863

[28] Schmidt M, Babcock L, Kurowski BG, Cassedy A, Sidol C, Wade SL. Usage patterns of an mHealth symptom monitoring app among adolescents with acute mild traumatic brain injuries. J Head Trauma Rehabil. 2022;37(3):134–43. 35125434 10.1097/HTR.0000000000000768 PMC9203862

[29] Trbovich AM, Howie EK, Elbin RJ, Ernst N, Stephenson K, Collins MW, et al. The relationship between accelerometer-measured sleep and next day ecological momentary assessment symptom report during sport-related concussion recovery. Sleep Health. 2021;7(4):519–25. 33933377 10.1016/j.sleh.2021.03.006

[30] Worthen-Chaudhari L, McGonigal J, Logan K, Bockbrader MA, Yeates KO, Mysiw WJ. Reducing concussion symptoms among teenage youth: Evaluation of a mobile health app. Brain Inj. 2017;31(10):1279–86. doi: 10.1080/02699052.2017.1332388 28665690 PMC5645232

[31] Sufrinko AM, Howie EK, Charek DB, Elbin RJ, Collins MW, Kontos AP. Mobile Ecological Momentary Assessment of Postconcussion Symptoms and Recovery Outcomes. J Head Trauma Rehabil. 2019;34(6):E40–8. doi: 10.1097/HTR.0000000000000474 30829823

[32] Lenaert B, Colombi M, van Heugten C, Rasquin S, Kasanova Z, Ponds R. Exploring the feasibility and usability of the experience sampling method to examine the daily lives of patients with acquired brain injury. Neuropsychol Rehabil. 2019;29(5):754–66. doi: 10.1080/09602011.2017.1330214 28562164

[33] Schoenfeld R, Drendel A, Ahamed SI, Thomas D. Longitudinal Assessment of Acute Concussion Outcomes Through SMS Text (ConText Study). Pediatr Emerg Care. 2022;38(1):e37–42. doi: 10.1097/PEC.0000000000002596 34986585

[34] Schlichter E, Lopez O, Scott R, Ngwenya L, Kreitzer N, Dangayach NS, et al. Feasibility of nurse-led multidimensional outcome assessments in the neuroscience intensive care unit. Crit Care Nurse. 2020;40(3):e1-8. 10.4037/ccn2020681 32476030

[35] Anthony CA, Peterson AR. Utilization of a text-messaging robot to assess intraday variation in concussion symptom severity scores. Clin J Sport Med. 2015;25(2):149–52. doi: 10.1097/JSM.0000000000000115 24905538

[36] Pacella M, Prabhu A, Morley J, Huang S, Suffoletto B. Postconcussive Symptoms Over the First 14 Days After Mild Traumatic Brain Injury: An Experience Sampling Study. J Head Trauma Rehabil. 2018;33(3):E31–9. doi: 10.1097/HTR.0000000000000335 28926480

[37] Wade SL, Sidol C, Babcock L, Schmidt M, Kurowski B, Cassedy A, et al. Findings from a randomized controlled trial of SMART: An eHealth intervention for mild traumatic brain injury. J Pediatr Psychol. 2023;48(3):241–53. 36565462 10.1093/jpepsy/jsac086 PMC10027050

[38] Suffoletto B, Wagner AK, Arenth PM, Calabria J, Kingsley E, Kristan J, et al. Mobile phone text messaging to assess symptoms after mild traumatic brain injury and provide self-care support: a pilot study. J Head Trauma Rehabil. 2013;28(4):302–12. doi: 10.1097/HTR.0b013e3182847468 23474882

[39] Shukla D, Devi BI, Agrawal A. Outcome measures for traumatic brain injury. Clin Neurol Neurosurg. 2011;113(6):435–41. doi: 10.1016/j.clineuro.2011.02.013 21440363

[40] Longo UG, Carnevale A, Massaroni C, Lo Presti D, Berton A, Candela V, et al. Personalized, predictive, participatory, precision, and preventive (P5) medicine in rotator cuff tears. J Pers Med. 2021;11(4):255. 33915689 10.3390/jpm11040255 PMC8066336

[41] Ahad AA, Sanchez-Gonzalez M, Junquera P. Understanding and Addressing Mental Health Stigma Across Cultures for Improving Psychiatric Care: A Narrative Review. Cureus. 2023;15(5):e39549. doi: 10.7759/cureus.39549 37250612 PMC10220277

[42] Duggal M, El Ayadi A, Duggal B, Reynolds N, Bascaran C. Editorial: Challenges in implementing digital health in public health settings in low and middle income countries. Front Public Health. 2023;10:1090303. doi: 10.3389/fpubh.2022.1090303 36703825 PMC9872111

[43] Christoforou AN, Armstrong MJ, Bergin MJG, Robbins A, Merillat SA, Erwin P, et al. An evidence-based methodology for systematic evaluation of clinical outcome assessment measures for traumatic brain injury. PLoS One. 2020;15(12):e0242811. doi: 10.1371/journal.pone.0242811 33315925 PMC7735614

[44] World Health Organization. How to use the ICF: A practical manual for using the International Classification of Functioning, Disability and Health (ICF). https://cdn.who.int/media/docs/default-source/classification/icf/drafticfpracticalmanual2.pdf?sfvrsn=8a214b01_4&download=true. 2013.

[45] McCulloch KL, de Joya AL, Hays K, Donnelly E, Johnson TK, Nirider CD, et al. Outcome Measures for Persons With Moderate to Severe Traumatic Brain Injury: Recommendations From the American Physical Therapy Association Academy of Neurologic Physical Therapy TBI EDGE Task Force. J Neurol Phys Ther. 2016;40(4):269–80. doi: 10.1097/NPT.0000000000000145 27576089

[46] Retzer A, Calvert M, Ahmed K, Keeley T, Armes J, Brown JM, et al. International perspectives on suboptimal patient-reported outcome trial design and reporting in cancer clinical trials: A qualitative study. Cancer Med. 2021;10(16):5475–87. doi: 10.1002/cam4.4111 34219395 PMC8366078

[47] Aiyegbusi OL, Roydhouse J, Rivera SC, Kamudoni P, Schache P, Wilson R, et al. Key considerations to reduce or address respondent burden in patient-reported outcome (PRO) data collection. Nat Commun. 2022;13(1):6026. doi: 10.1038/s41467-022-33826-4 36224187 PMC9556436

[48] Global strategy on digital health 2020-2025. Genève, Switzerland: World Health Organization. 2021.

[49] International Classification of Health Interventions (ICHI Beta-3 Reference Guide). World Health Organization. 2020. https://mitel.dimi.uniud.it/ichi/docs/ICHI%20Beta-3%20Reference%20Guide.pdf

[50] Bashshur R, Shannon G, Krupinski E, Grigsby J. The taxonomy of telemedicine. Telemed J E Health. 2011;17(6):484–94. doi: 10.1089/tmj.2011.0103 21718114

[51] The World Bank Group. World Bank Country and Lending Groups. https://datahelpdesk.worldbank.org/knowledgebase/articles/906519-world-bank-country-and-lending-groups. 2024. 2024 July 10.

