# Supplementary material for: Community-based digital mental health interventions for traumatic brain injury patients: A scoping review
Source: PLOS Ment Health. 2025 Aug 19;2(8):e0000397. doi: 10.1371/journal.pmen.0000397 (PMC12798249; doi:10.1371/journal.pmen.0000397)
Supplement: S1 Table — (S1_Table.DOCX) [file pmen.0000397.s001.docx]

| **Supplementary File 1:** Full justification of PCC criteria employed in article screening |
| --- |
| ***Population/Participants***  Studies were only eligible for inclusion in this review if their primary aim related to the development, implementation, or validation of technologies contributing to the provision of remote digital mental health services of discharged paediatric and adult patients following TBI of any severity, whether directly or via proxy (family members/relatives, caregivers, and guardians). Studies of mixed neuropathological cohorts (e.g., traumatic brain injury and stroke, traumatic brain injury and post-traumatic stress disorder) were included.  ***Concept***  In this review, we adopt the World Health Organization’s (WHO) definition of ‘digital health’: “*the field of knowledge and practice associated with the development and use of digital technologies to improve health [...] expand[ing] the concept of eHealth to include digital consumers, with a wider range of smart and connected devices. It also encompasses other uses of digital technologies for health such as the Internet of Things, advanced computing, big data analytics, artificial intelligence including machine learning, and robotics*”.[(48)](https://paperpile.com/c/HKKXi5/z8Dle)  Interchangeably with terms ‘solution’, ‘platform’ and ‘technologies’, we additionally adopt the WHO’s definition of ‘health intervention’: “*A health intervention is an act performed for, with or on behalf of a person or a population whose purpose is to assess, improve, maintain, promote or modify health, functioning or health conditions.*”[(49)](https://paperpile.com/c/HKKXi5/8ZkRc) In the context of this review, we further defined *a priori* that a DMHI as any digital health service or platform supporting the exchange of data from the patient as to their current mental welfare status, either in the form of patient-generated health data (PGHD), that is, self-reported, such as patient-reported outcome measures (PROMs), or clinician-derived health data (CDHD), that is, information acquired through clinician-led assessment or interview, either with the patient directly, or via their nominated proxy.  With respect to PROMs of interest, we included any instruments comprising items relating to affective/emotional, behavioural, or psychological well-being, excluding any solely assessing cognition (i.e., attention and working memory, communication, reasoning, coordination).  Herein, we refer to synchronicity as the temporal aspect of the encounter between clinician and patient. Synchronous DMHIs function to collect data in real time, often allowing direct contact between clinician and patient through sensor-, text-, voice-, or video-based technology. Asynchronous DMHIs collect data by store-and-forward techniques—data are gathered, stored, and transmitted for later review by the clinician at two independent time-points; that is, they do not interact in real time.[(50)](https://paperpile.com/c/HKKXi5/69fKd)  Finally, we define “follow-up” as any attempt to monitor, assess, communicate, or liaise with a patient, or their proxy, from the point of hospital discharge, for the benefit of furthering their health and well-being, research, or injury surveillance.  ***Context***  DMHI implementations in any global setting. We utilise high-income country and low- and middle-income country classifications as defined by the World Bank.[(51)](https://paperpile.com/c/HKKXi5/Q4OB6)  We define “remote” as, at the time of information exchange, the patient was in a community-based setting (e.g., home, residential treatment facility, community care provider or outpatient clinic, or another public setting) at a distance from the clinical team primarily responsible for their care.[(4)](https://paperpile.com/c/HKKXi5/d4hl8) Articles with mixed settings, i.e., transitioning from the emergency department to a community setting during the study, were also included. |
