# Supplementary material for: Community-based digital mental health interventions for traumatic brain injury patients: A scoping review
Source: PLOS Ment Health. 2025 Aug 19;2(8):e0000397. doi: 10.1371/journal.pmen.0000397 (PMC12798249; doi:10.1371/journal.pmen.0000397)
Supplement: S2 Table — (S2_Table.DOCX) [file pmen.0000397.s002.docx]

| **Supplementary File 2: Search strategies across selected databases** |
| --- |

| **OVID Medline** | |
| --- | --- |
| 1 | (TBI or traumatic brain injur* or brain injur* or head injur* or concuss* or brain traum* or head traum*).mp. [mp=title, book title, abstract, original title, name of substance word, subject heading word, floating sub-heading word, keyword heading word, organism supplementary concept word, protocol supplementary concept word, rare disease supplementary concept word, unique identifier, synonyms, population supplementary concept word, anatomy supplementary concept word] |
| 2 | exp Brain Injuries, Traumatic/ |
| 3 | exp Craniocerebral Trauma/ |
| 4 | exp Outcome Assessment, Health Care/ |
| 5 | exp Patient Generated Health Data/ |
| 6 | exp "Surveys and Questionnaires"/ |
| 7 | exp Ecological Momentary Assessment/ |
| 8 | outcome measure*.mp. [mp=title, book title, abstract, original title, name of substance word, subject heading word, floating sub-heading word, keyword heading word, organism supplementary concept word, protocol supplementary concept word, rare disease supplementary concept word, unique identifier, synonyms, population supplementary concept word, anatomy supplementary concept word] |
| 9 | exp Mental Health/ |
| 10 | exp Behavioral Symptoms/ |
| 11 | (depression or anxiety or anger or post-traumatic stress disorder* or post traumatic stress or PTSD or anhedonia or low mood or anxiety disord* or irritabil* or letharg* or nervous* or emotional).mp. [mp=title, book title, abstract, original title, name of substance word, subject heading word, floating sub-heading word, keyword heading word, organism supplementary concept word, protocol supplementary concept word, rare disease supplementary concept word, unique identifier, synonyms, population supplementary concept word, anatomy supplementary concept word] |
| 12 | exp Mental Disorders/ |
| 13 | exp Digital Health/ |
| 14 | exp Telemedicine/ |
| 15 | exp Telecommunications/ |
| 16 | 1 or 2 or 3 |
| 17 | 4 or 5 or 6 or 7 or 8 or 13 or 14 or 15 |
| 18 | 9 or 10 or 11 or 12 |
| 19 | 16 and 17 and 18 |

| **OVID Embase** | |
| --- | --- |
| 1 | exp traumatic brain injury/ |
| 2 | (TBI or traumatic brain injur* or brain injur* or head injur* or concuss* or brain traum* or head traum*).mp. [mp=title, abstract, heading word, drug trade name, original title, device manufacturer, drug manufacturer, device trade name, keyword heading word, floating subheading word, candidate term word] |
| 3 | exp outcome assessment/ |
| 4 | exp health survey/ |
| 5 | exp ecological momentary assessment/ |
| 6 | exp patient-reported outcome/ |
| 7 | outcome measure*.mp. [mp=title, abstract, heading word, drug trade name, original title, device manufacturer, drug manufacturer, device trade name, keyword heading word, floating subheading word, candidate term word] |
| 8 | exp mental health/ |
| 9 | exp mental disease/ |
| 10 | (depression or anxiety or anger or post-traumatic stress disorder* or post traumatic stress or PTSD or anhedonia or low mood or anxiety disord* or irritabil* or letharg* or nervous* or emotional).mp. [mp=title, abstract, heading word, drug trade name, original title, device manufacturer, drug manufacturer, device trade name, keyword heading word, floating subheading word, candidate term word] |
| 11 | exp digital health/ |
| 12 | exp telemedicine/ |
| 13 | exp telehealth/ |
| 14 | (mhealth or mobile health or ehealth).mp. [mp=title, abstract, heading word, drug trade name, original title, device manufacturer, drug manufacturer, device trade name, keyword heading word, floating subheading word, candidate term word] |
| 15 | exp telecommunication/ |
| 16 | 1 or 2 |
| 17 | 3 or 4 or 5 or 6 or 7 |
| 18 | 8 or 9 or 10 |
| 19 | 11 or 12 or 13 or 14 or 15 |
| 20 | 16 and 17 and 18 and 19 |

| **Web of Science** |
| --- |
| ((ALL=(TBI or traumatic brain injur* or brain injur* or head injur* or concuss* or brain traum* or head traum*)) |
| AND ALL=(outcome assess* or outcome measure* or patient*reported outcome measure* or PROM* or survey or questionnaire or ecological momentary assessment or EMA)) |
| AND ALL=(mental health or mental disorder* or mental illness* or behavio*ral symptom* or depression or anxiety or anger or post*traumatic stress disorder or PTSD or anhedonia or low mood or anxiety or irritabil* or letharg* or nervous* or emotional)) |
| AND ALL=(digital health or mhealth or mobile health or telemed* or telehealth* or ehealth) |

| **Scopus** |
| --- |
| (TBI or traumatic brain injur* or head injur* or concuss* or brain traum* or head traum*) |
| AND (outcome assess* or patient*reported outcome measure or prom or patient generated health data or PGHD or survey or questionnaire or ecological momentary assessment or EMA) |
| AND (mental health or mental illness* or mental disorder* or behavio*ral symptoms or depression or anxiety or anger or post*traumatic stress disorder or ptsd or anhedonia or low mood or anxiety disorder* or irritabil* or nervous* or emotion*) |
| AND (digital health or mhealth or ehealth or telemed* or telehealth*) |

| **EBSCO PsycInfo** |
| --- |
| 1. TBI or traumatic brain injur* or brain injur* or head injur* or concuss* or brain traum* or head traum* |
| 1. outcome assess* or outcome measure* or patient*reported outcome measure* or PROM* or survey or questionnaire or ecological momentary assessment or EMA |
| 1. mental health or mental disorder* or mental illness* or behavio*ral symptom* or depression or anxiety or anger or post*traumatic stress disorder or PTSD or anhedonia or low mood or anxiety or irritabil* or letharg* or nervous* or emotional |
| 1. digital health or mhealth or mobile health or telemed* or telehealth* or ehealth |

| **Global Health** |
| --- |
| TBI or traumatic brain injur* or brain injur* or head injur* or concuss* or brain traum* or head traum* |
| outcome assessment* or outcome measure* or patient-reported outcome measure* or PROM* or patient generated health data or PGHD |
| mental health or mental disorder* or mental illness* or behavio*ral symptom* or depression or anxiety or anger or post*traumatic stress disorder or PTSD or anhedonia or low mood or anxiety or irritabil* or letharg* or nervous* or emotional |
| digital health or telemed* or mhealth or telehealth or ehealth |
