# Supplementary material for: Community-based digital mental health interventions for traumatic brain injury patients: A scoping review
Source: PLOS Ment Health. 2025 Aug 19;2(8):e0000397. doi: 10.1371/journal.pmen.0000397 (PMC12798249; doi:10.1371/journal.pmen.0000397)
Supplement: S3 Table — (S3_Table.DOCX) [file pmen.0000397.s003.docx]

**Supplementary File 3: Charting proforma of all articles retrieved (n = 23)**

| Author | Year | Title | Article Type & Study Design | Country | Aims / objectives / purpose | Pathology | TBI Description | Reported comorbidities / pre-existing history | Time since injury category | Time since injury description | Population category | Demographic stats | Total population (n) and description | TBI sub-population (n) | Data requester | Modality | Patient (or proxy) location | Digital mental health session description (number of sessions, frequency) | Synchronicity of digital mental health solution | Use of proxy and description | Mental health clinician- or patient-reported outcome measures utilised | Key findings |
| --- | --- | --- | --- | --- | --- | --- | --- | --- | --- | --- | --- | --- | --- | --- | --- | --- | --- | --- | --- | --- | --- | --- |
| Riggall, et al. | 2024 | Caregiver and family functioning after pediatric disorder of consciousness: telephone-based outcome assessment. | Original - Descriptive | USA | To examine initial feasibility/utility of a telephone-administered measure in describing impact of child health on caregiver/family functioning in patients with a history of a disorder of consciousness (DoC) due to severe-acquired brain injury (ABI). | TBI and non-traumatic brain injury (i.e., ABI) | Not reported | Not explicitly reported | 12mo< | 1 - 18 years (Avg = 5.3 SD = 4.2) | Caregivers of paediatric patients | 78% mothers, 37% female patients | 41 caregivers, 33 ABI patients (n=18 TBI) | 18 | Master's level trained administrator | Telephony | Commuity | Once | Sync | No | PedsQL-FIM, GOS-E Peds | Within this relatively small convenience sample, results indicate the PedsQL-FIM administered via telephone is feasible and useful in describing the impact of child health on caregiver/family functioning long after DoC associated with ABI. Future studies are needed to understand factors contributing to caregiver/family functioning to inform targeted interventions. Family functioning was lowest in Daily Activities and highest in Family Relationships. Relative to caregivers of patients with mild-severe ABI, caregivers reported lower caregiver/family functioning. Correlations were moderate between child functioning and caregiver/family functioning on some PedsQL-FIM scales. |
| Shukla, et al. | 2023 | Validity of Glasgow outcome scale-extended (GOSE) mobile application for assessment of outcome in traumatic brain injury patients | Original - Quasi-experimental | India | To develop the Glasgow Outcome Scale-Extended (GOSE) mobile application and examine the validity of the application against GOSE scoring based on traditional interview method. | TBI | Mild, Moderate, Severe | Not explicitly reported | 12mo< | Up to 6 years (median 6mo) | Adults |  | 102 | 102 | Clinician 'rater' | Smartphone app | Outpatient clinic | Once | Sync | Yes - clinician | Translated GOS-E (Kannada) | The GOSE mobile application can measure GOSE Score similar to the traditional interview method. This application may help fasten the process of assessing outcome in TBI patients in clinical practice and in research. GOS-E remains limited in describing overall change post injury, and hence it does not attribute to disability caused by brain injury alone. Both raters also familiar with GOS-E scoring, possibly attributing to high agreement between mobile app and traditional GOS-E. |
| Wade, et al. | 2023 | Findings from a randomized controlled trial of SMART: An Ehealth intervention for mild traumatic brain injury | Original - Experimental (Two-arm, parallel design RCT) | USA | To examine the efficacy of the SMART (Self-Management After Recent Traumatic brain injury) program and potential moderators. | TBI | Mild | Not explicitly reported | <1wk | Average 1.6 days (SD 2.3) | Adolescents | Avg 14.2y (SD 1.8), 47.9% F, 76.1% white, 23.9% black/african american | 71 (n=36 usual care/control, n=35 mHealth) | 71 | Research coordinator | Smartphone app | ED / Community | Daily for 4 weeks | Async | No | PCSI | SMART program utilization varied widely, with the frequency of symptom monitoring ranging from 1 to 34 times (M = 9.66, SD = 9.76). Given the study design, we were unable to examine the unique and additive contributions of the symptom monitoring and learning module components of the SMART intervention. Effectiveness of the SMART app varied based on preinjury coping styles and resilience, underscoring the potential need to tailor treatments to individual characteristics. |
| Nabasny, et al. | 2022 | Neurobehavioral Symptoms and Heart Rate Variability: Feasibility of Remote Collection Using Mobile Health Technology | Original - Quasi-experimental (prospective repeated measures) | USA | To determine the covariance of heart rate variability (HRV) and self-reported neurobehavioral symptoms after traumatic brain injury (TBI) collected using mobile health (mHealth) technology. | TBI and healthy | Mild, Moderate, Severe | Not explicitly reported | Not reported | Not explicitly reported | Adults with lifetime history of TBI, and adults with no history of brain injury |  | 64 (12 healthy) | 40 | Research team | Mixed (SMS text link to RedCap web platform) | Community | Once daily or every other day over two weeks, at either scheduled or random times | Async | No | BAST (mHealth short-form version) | Compliance in our study was comparable to past studies examining EMA in TBI. It is generally feasible for community-dwelling adults with and without TBI to use a commercially available wearable device to capture daily HRV measures and to complete a short, electronic self-reported neurobehavioral symptom measure for a two-week period. Though participants overwhelmingly indicated that using the mHealth technology was easy, one participant made an important observation that they were a “tech savvy person” and that “someone who is not may have needed more detailed instruction.” Given the high education level, the likely bias toward more tech-savvy adults by recruiting completely online, and the relatively small proportion of individuals with more severe TBI in our study, we advise caution in assuming that this reported ease of use would necessarily generalize to those with lower tech-literacy and/or more severe cognitive disability post-injury. |
| Schmidt, et al. | 2022 | Usage Patterns of an mHealth Symptom Monitoring App Among Adolescents With Acute Mild Traumatic Brain Injuries | Original - Quasi-experimental | USA | To understand usage patterns of SMART mHealth app (Self-Monitoring Activity Regulation and Relaxation Treatment) among adolescents with acute mild traumatic brain injuries (mTBI) and identify individual characteristics that influenced app usage. | TBI | Mild | Comorbid ADHD, Anxiety, Depression | <1mo | Within two weeks | Adolescents | 47.2%F, mean age 14.2 (1.8), 65.8% identified as white | 34 | 34 | Not reported | Mixed (Gamified mobile learning environment (GMLE) - mHealth app / website) | ED / Community | Once daily until symptom resolution | Async | No | PCSS | Adolescents of higher socioeconomic status and those who manage their emotions using active engagement spent more time on both components of the SMART program. |
| Schoenfeld, et al. | 2022 | Longitudinal Assessment of Acute Concussion Outcomes Through SMS Text (ConText Study) | Original - Observational (prospective Cohort pilot) | USA | This study uses SMS text messaging (a mobile health [mHealth] tool) to report patient symptoms. We aim to better characterize mTBI recovery and hypothesize that this mHealth tool will have high retention rates and correlate with a conventional means of assessing symptoms, the Post-Concussion Symptom Inventory (PCSI). | TBI | Mild | Not explicitly reported | <1wk | Within 48h of injury | Paediatric and adolescent patients | 58% F, age range 8 - 18. | 31 | 31 | Not reported | SMS | ED / Community | Proprietary mHealth measure delivered Day 0, then once every 3 days for first 21 days, then once a week for 6 weeks. PCS and PCSI only delivered Day 0 (in ED). | Async | No | Pain Catastrophizing Scale (PCS), Post-Concussion Symptom Inventory (PCSI), proprietary mHealth measure adapted from PCSI with additional measures including symptom resolution and return to activity | In this study, we demonstrate that SMS text messaging is a valid and easy-to-use method of reporting pediatric mTBI symptoms. Participants reported that using text messaging to report their symptoms was convenient and user friendly, while still being able to accurately describe their pain experience. However, the retention rate throughout the entire study was not as high as expected. After the resolution of mTBI symptoms, participation in the study dropped. There are several potential explanations for this. SMS messaging is inherently intrusive, and participants may not have seen the value in monitoring symptoms after resolution. Unfortunately, our study may have failed to capture the reemergence of symptoms as the patient steps up his or her activity level. The limitation of answering each question separately combined with the time required for completion of our mHealth tool (approximately 5–10 minutes) could have also contributed to the response rates. A more streamlined tool may be more successful for long-term retention. However, given that most subjects continued to track symptoms until resolution, may provide a useful tool for providers in the Acute Care setting. Mobile health tools (either SMS or smartphone) could be used to track symptoms and encourage follow up with the primary care team based on preprogrammed metrics (eg, failure to achieve symptom resolution by a set time point) and provide patients with a record of postinjury symptoms to share with providers on follow up. This pilot study demonstrates that this tool is a valid and easy-to-use method of reporting pediatric mTBI symptoms—it replicates and identifies novel findings. Our results suggest that there may be a relationship between balance and the manifestation of somatic symptoms. Retention rates were lower than predicted, indicating that text messaging may not be the ideal format in this population. Text messaging may still have other applications for short-term communication/symptom measurement. |
| Trbovich, et al. | 2021 | The relationship between accelerometer-measured sleep and next day ecological momentary assessment symptom report during sport-related concussion recovery | Original - Quasi-experimental (prospective repeated measures) | USA | To use actigraphy and ecological momentary assessment (EMA) to examine the relationship between sleep parameters and next day symptoms. | TBI | Mild | Depression, anxiety, ADHD | <1wk | Within 72 of injury | Adolescents | Avg 15.35y +/- 2.09; 47.1% F | 17 | 17 | Not reported | Smartphone app | Community | Three times daily - 7am/9am, 3pm, 9pm - around school times, until recovery. | Async | No | PCSS | This study sought to overcome limitations on prior research examining the effects of sleep following SRC by objectively measuring sleep with accelerometers and integrating real-time or EMA symptom report. |
| Schlichter, et al. | 2020 | Feasibility of Nurse-Led Multidimensional Outcome Assessments in the Neuroscience Intensive Care Unit | Original - Observational | USA | To determine the feasibility of multidimensional outcome assessments in neurocritical care patients post-discharge- wanted to assess whether it was practical and efficient to conduct comprehensive follow-up evaluations on patients who had been treated in a neuroscience intensive care unit (NICU) after they had been discharged using technologies | TBI and non-traumatic brain injury (i.e., ABI) | Not reported | Not specifically described, though anxiety and depression are mentioned in outcome measures | 3mo-6mo | 4.4 months after admission (mean) | Adults | Mean: 59.5y F 45.3% | 1324 | 218 | Not reported | Telephony | Hospital (NICU) / Community | Single follow-up call | Sync | Yes- patients or caregiver | EQ-5D-5L (5-level EQ-5D) | Successfully contacted 76.4% of patients (loss to follow-up rate of 23.6%). The multidimensional outcome assessment was feasible and efficient. Completed assessments took an average of 21 minutes 54 seconds. Successfully gathered data across multiple domains (physical, cognitive, quality of life). |
| Lenaert, et al. | 2019 | Exploring the feasibility and usability of the experience sampling method to examine the daily lives of patients with acquired brain injury | Original - Observational (longitudinal observational) | The Netherlands | To investigate the feasibility of using experience sampling method (ESM) in individuals with acquired brain injury (ABI), to explore the usability of ESM data on a clinical level, by illustrating the interactions between person, environment, and affect | TBI or stroke | Not reported | Not explicitly reported | Not reported | Not reported | Adults | Of whole pop, n=17, Average age (SD, range) = 44.2y (14.5, 18–65), Sex (F) = 53% | 17 | 8 | Not reported | Custom device (Custom touchscreen electronic device 'PsyMate') | Community | 10 instances delivered daily at semi-random scheduled times over 6 days | Async | No | Positive & Negative Affect Schedule (PANAS) | This study illustrates the potential of ESM to identify complex person–environment dynamics after ABI, while generating understandable and easy to use graphical feedback. Average response rate of 71.18% (n = 726) following delivery of 1020 prompts 98.76% of prompted reports were completed, with an average of 42.7 questionnaires answered (range 28–57) There were no dropouts reported, with the method experienced as user-friendly The device was reported to have little influence on their activities or social contacts (average = 2.00/7, SD 1.16). There were little to no difficulties reported when using the device (average = 1.77/7, SD 1.36), with the device not experienced as burdensome (average = 2.08/7, SD 1.19) The amount of beeps were not seen as much of a burden (average = 2.08/7, SD 1.19) |
| Sufrinko, et al. | 2019 | Mobile Ecological Momentary Assessment of Post-concussion Symptoms and Recovery Outcomes. | Original - Quasi-experimental (prospective repeated measures) | USA | To evaluate mobile ecological momentary assessment (mEMA) as an approach to measure sport-related concussion (SRC) symptoms, explore the relationships between clinical outcomes and mEMA, and determine whether mEMA was advantageous for predicting recovery outcomes compared to traditional symptom report. | TBI | Mild | Anxiety, depression | <1wk | Within 72 hours | Adolescents | Mean age 15.35 (1.98), 40% F. | 20 | 20 | Not reported | Smartphone app | Outpatient concussion clinic / community | Three instances of assessment at predetermined fixed time blocks (morning, afternoon, evening) daily until second follow-up or medical clearance (whichever first) | Async | No | PCSS | 90% of participants responded to mEMA prompts with an overall response rate of 52.4% (n = 1155 prompts)  Average response rate of 50.4% (SD 29.3) per participant responded to throughout the study, with a range of 5.4% to 95.2%  Average prompts received n = 64, range 19–173)  There was no correlation between number of prompts received and the response rate (Spearman rho = 0.08, p = 0.77)  Participants were less likely to respond as days since injury increase (OR = .91, 95% CI: 0.87-0.94, p < 0.001)  Response rate differed by age with older participants less likely to respond (OR = 0.56, 95% CI: 0.34-0.93, p = 0.026).  There was no association between response rate and time of day (morning = 50.1%, afternoon = 52.9%, evening = 49.8%; p = 0.411)  There was no difference in response rate for initial symptom burden (OR = 0.97, 95% CI: 0.92-1.03; p = 0.354) |
| Juengst, et al. | 2019 | Variability in daily self-reported emotional symptoms and fatigue measured over eight weeks in community dwelling individuals with traumatic brain injury. | Original - Descriptive (prospective pilot) | USA | To investigate within-person variability in daily self-reported emotional and fatigue symptoms in individuals with chronic TBI, and identify factors associated with high within-person variability. | TBI | Mild, Moderate, Severe | Anxiety, depression | 12mo< | 2 - 27 years after injury | Adults | Mean 38.3y (12.7, range 22-60); 72% F, 83% white. | 18 | 18 | Not reported | Smartphone app | Communiuty | Daily assessments for 8 weeks (56 time points total) | Async | No | PANAS, PHQ-2, GAD-2 | Significant temporal within-person variability occurred for all measures. High variability was associated with more symptom reporting versus Low variability, and variability was associated with sex (High variability: 88% women; Low variability 90% men). Symptom measurement at a single time point among adults with chronic TBI may not capture day-to-day symptom fluctuation and may misidentify individuals in need of intervention. Assessing symptom profiles over time to capture temporal and individual variability may provide a more ecologically valid measure for managing long-term symptoms after TBI. |
| Vaca, et al. | 2019 | Long-term follow-up of pediatric head trauma patients treated at Mulago National Referral Hospital in Uganda | Original - Descriptive | Uganda | To describe the use of a novel method of telephone surveys to conduct the first-ever long-term follow-up in Uganda to elucidate the outcomes of pediatric head trauma patients treated at the national referral hospital | TBI | Mild, Moderate, Severe | Not explicitly reported | 12mo< | 1.48y post-injury | Paediatric | Median age (range) = 6y (0.17–17) Sex (F) = 29% | 142 | 142 | Local research assistants | Telephony | Community | Once at 1y or 2y post-discharge | Sync | Yes (wholly) - parent | GOSE-Peds and Proprietary quality of life interview including psychosocial function (Friendly, responsible, dispalys emotion, behaves with parents) | This first-ever long-term follow-up of pediatric head trauma patients in Uganda confirmed the feasibility of a novel phone follow-up method for patients throughout Uganda. The current study lays the groundwork for phone follow-up in low- and middle-income countries as a viable way to obtain outcome data. Average call duration 20 min. With up to 5 contact attempts, achieved a 61% response rate (of initial n = 232 patients identified), representing 67% of patients receiving treatment and discharge in 12 months with a phone number on file. Suggested as a suitable alternative for home visits for a large referral hospital |
| Xu, et al. | 2018 | Life After the Neurosurgical Ward in Sub-Saharan Africa: Neurosurgical Treatment and Outpatient Outcomes in Uganda | Original - Descriptive | Uganda | To describe the use of phone surveys developed and conducted in the 40 participants’ language to assess mortality, neurological outcomes, and follow-up health care | TBI, spina bifida, hydrocephalus, brain tumours | Mild, Moderate, Severe | Not explicitly reported | 12mo< | 1.53 years post-injury/disease (all pathology, n=870) | Adult and paediatric | All pathology (n=870) and reachable by telephone: Median age = 26y  Sex (F) = 19% | 1167 | 740 | Local research assistants | Telephony | Community | Once | Sync | Use of proxy (designated family member) in some instances to initiate contact with patient or collection of demographics | GOS-E or pediatric version (GOSE-peds)  General survey items pertaining to: quality of life (continuing physical deficits), activities of daily living (ADLs), ability to speak and follow commands, perform household chores, school and work function, psychosocial function, and subjective return to baseline functional status | Phone surveys captured data on patients in whom nearly one-half would be lost to subsequent health care. Utilizing telephone, there was a 74.5% response rate (n = 870) Of those reached, no patient refused telephone assessment 70% of those who survived pre-discharged (n = 1167) had a phone number on file. |
| Pacella, et al. | 2018 | Postconcussive symptoms over the first 14 days after mild traumatic brain injury: an experience sampling study | Original - Observational | USA | To examine changes in postconcussive symptoms (PCS) over the acute postinjury recovery period, focusing on how daily PCSs differ between mild traumatic brain injury (mTBI) and other injury types using an experience sampling method conducted through text-messaging. | mTBI, head injury without TBI, non-head injury trauma controls | Mild | Co-morbid PTSD assesed prior | <1wk | Within 24h of injury, symptoms tracked for 14 days post-injury | Adults | 18-55 years, mean age 33 years, 52% F | 108 | 39 | Not reported | SMS | ED / Community | 3/day with 4hr response window for 14 days | Async | No | RPQ, PTSD Checklist-Civilian (PCL) in advance | Demonstrated the effectiveness of an SMS-based Experience Sampling Method (ESM) for monitoring postconcussive symptoms (PCS) in trauma patients over 14 days. The technology enabled frequent, real-time data collection in participants' natural environments, resulting in high response rates. Key findings revealed that patients with head injuries, regardless of meeting mild traumatic brain injury (mTBI) criteria, initially reported higher rates of PCS but showed faster recovery compared to trauma controls. The SMS system allowed for the detection of nuanced differences in symptom patterns across injury types and over time, which may not have been captured by traditional assessment methods |
| Worthen-Chaudhari, et al. | 2017 | Reducing concussion symptoms among teenage youth: Evaluation of a mobile health app | Original - Quasi-experimental (Non-randomised open label clinical trial) | USA | To evaluate whether a mobile health application that employs elements of social game design could compliment medical care for unresolved concussion symptoms. | TBI | Mild | Not explicitly reported | <12mo | 3 weeks to 12 months post injury | Adolescents |  | 39 | 39 | Not reported | Smartphone app | Outpatient clinic / Community | One logged activity (i.e., any in-app action such as reporting a 'symptom battled' and how severe the battle was (e.g., sadness)) per day for 5 days each week, for a target dose of 15 logged activities over first 3 weeks between pre and post-test. | Async | No | SCAT-3 | Mobile apps incorporating social game mechanics and a heroic narrative may promote health management among teenagers with unresolved concussion symptoms. Study limitations include small sample size, single testing site and lack of blinding or random assignment to treatment groups. In addition, we did not address technical barriers to participation or potentially clinically important qualitative differences between cohorts (e.g. mechanism of injury, types of concussion symptoms). Finally, findings may not be generalizable to youth with ADHD or adults with concussion, as representatives of these populations were not studied. These findings suggest that tapping into existing habits, such as mobile device and social network activity, with a gamified app is a feasible and potentially effective way to facilitate medical care among youth with concussion. |
| Thibault-Halman, et al. | 2017 | Early telephone follow-up for traumatic brain injury patients using the Rivermead Post-Concussion Symptoms Questionnaire | Abstract - Descriptive | Canada | To examine the frequency and severity of common post-TBI symptoms, as assessed by the RPCQ | TBI | Mild, Moderate | Not explicitly reported | 1wk-1mo | 2 weeks post-discharge | Adult | Not reported | 46 | 46 | Rehab-based nurse practitioner | Telephony | Community | Once | Sync | No | Rivermead Post-concussion Symptoms Questionnaire | 100% of patients were reached, where post-TBI symptoms in at least one domain (emotional, somatic, cognitive) remained present in 100% of cases  Referral for additional formal assessment, symptom management, and advice was provided in 37% of cases. The RPCQ, by telephony, is a low-cost structured evaluative tool  which highlights needs and provides guidance for patients and caregivers; it also seems effective in identifying those who may require  formal clinical assessment. |
| Wiebe, et al. | 2016 | Ecologic Momentary Assessment to Accomplish Real-Time Capture of Symptom Progression and the Physical and Cognitive Activities of Patients Daily Following Concussion | Correspondence - Descriptive | USA | To determine the feasibility of EMA following youth concussion, gather real-time reports of cognitive and physical activity, and compare objective measures with real-time reported symptoms among youth during recovery after concussion | TBI | Mild | Not explicitly reported | <1wk | 6 days after injury (median, IQR 3-10) | Paediatric | Median age 15y (13-16); 47%F. | 36 | 36 | Not reported | Smartphone app (iPod Touch) | Community | Several times daily for 2 weeks | Async | No | PCSS | n = 28 (82%) responded to more than 80% of symptom questionnaire prompts. This study established that ecological momentary assessment provides a feasible approach to capture objective measures of physical activity and reports of cognitive activity and symptoms in real time in pediatric patients. In this pilot sample, we found that cognitive rest and physical activity on a given day corresponded with lower symptom severity on the subsequent 2 days. However, of interest, most patients were asymptomatic after 2 weeks of follow-up regardless of activity levels during that period. |
| Juengst, et al. | 2015 | Pilot feasibility of an mHealth system for conducting ecological momentary assessment of mood-related symptoms following traumatic brain injury | Original - Quasi-experimental (pilot study of prospective repeated measures design) | USA | To assess pilot feasibility and validity of a mobile health (mHealth) system for tracking mood-related symptoms after traumatic brain injury (TBI). | TBI | Mild, Moderate, Severe | Not explicitly reported | 12mo< | 5.2y (+/- 3.6) avg | Adults |  | 20 community dwelling adults with TBI | 20 | Not reported | Smartphone app | Community | 7 measures daily in a preferred 3-hr window over 8-week period, bi-weekly telephone | Async | No | PHQ-9; GAD-7; PHQ-2; PANAS; 7-pt fatigue scale | EMA conducted via smartphone demonstrates initial feasibility among adults with TBI and presents numerous opportunities for long-term monitoring of mood-related symptoms in real-world settings. Daily assessments took less than 2 minutes to complete. 73.4% assessments completed as scheduled; 79.8% completed as a whole 6.3/7 (SD 0.8) patient satisfaction with iPerform smartphone application assessment. From the TUQ, 6.2/7 (SD 0.8) reported ease of use, 4.3/7 (SD 1.7) for reliability and 5.5/7 (SD 1.1) for satisfaction with iPerform Low reliability thought to be due to technical problems faced by participants throughout study (notification receipt errors or application crashing and/or not submitting assessment) High correlations with standard telephone-interview supporting validity of smartphone-based mood-related EMA in this population |
| Anthony, et al. | 2015 | Utilization of a text-messaging robot to assess intraday variation in concussion symptom severity scores | Original - Observational | USA | To determine the amount of within-day variation of Concussion Symptom Severity Scores (CSSSs) in athletes with a clinically diagnosed concussion. | TBI | Mild | Not explicitly reported | 1wk-1mo | Not reported time of injury- mean follow-up of 23.9 days | Adolescents and young adults (14-22 years old) | 14-22 years old | 14 | 14 | Not reported | SMS (text-messaging robot) | Community | 5/day (30 day study period or until study participant responded for 7 consecutive days with PCSSS of 0) | Async | No | Post Concussion Symptom Score (PCSS), Concussion Symptom Severity Score (CSSS), Sport Concussion Assessment Tool 2 (SCAT2) Symptom Severity Score | The study used a 'text-messaging robot' (SMS) to assess intraday variation in Concussion Symptom Severity Scores (CSSS) among 14- to 22-year-old athletes with clinically diagnosed concussions. Participants received text messages five times daily to complete the Post Concussion Symptom Score (PCSS), from which the CSSS was derived. The study found significant within-day variation in reported symptoms, with a mean CSSS of 29.9 and a mean within-day standard deviation of 8.3. These findings challenge the validity of using infrequent CSSS assessments for tracking concussion severity and recovery over time. |
| Suffoletto, et al. | 2013 | Mobile phone text messaging to assess symptoms after mild traumatic brain injury and provide self-care support: a pilot study. | Original - Experimental (RCT) | USA | To examine whether patients with mild traumatic brain injury (mTBI) receiving text messaging-based education and behavioral support had fewer and less severe post-concussive symptoms than those not receiving text message support, and to determine the feasibility of using text messaging to assess daily symptoms and provide support to patients with mTBI | TBI | Mild | Not explicitly reported | Not reported | Not reported | Adults | Average age (SD) = 30 (9) Sex (F) = 56% | 43 (n=18 intervention, 25 control) | 43 | Blind examiner | SMS | Community | 14 days of timed SMS symptom assessments: 9am headache, 1pm difficulty conceentrating, 5pm irritiability or anxiety; derived from RPQ | Async | No | RPQ-derived brief symptom assessments | Those receiving the text messaging–based education and support had fewer and less severe postconcussive symptoms than the controls but none of the differences reached statistical significance. Further evaluation of more robust mobile interventions and larger sample of participants are still needed. 84% (n = 36) completed 14-day SMS follow-up  93% felt that messaging system was useful to help them self-manage and understand symptoms  Over 14 days, 74% completed 9 am headache assessment, 96% completed 1 pm difficulty concentrating assessment, and 97% completed 5 pm irritability/anxiety assessment  Among completed assessments, between 49% and 54% completed <1h, and 29% to 54% completed <5 min |
| Smith, et al. | 2012 | Enhancing behavioral health treatment and crisis management through mobile ecological momentary assessment and SMS messaging | Original - Descipritve | USA | To assess the utility of mobile health (mHealth) technologies, including personal digital assistant-based ecological momentary assessment and two-way interactive text (SMS) messaging, for providing treatment feedback to clinicians, encouraging and motivating veterans throughout treatment, and monitoring participants for relapse after treatment discharge. | TBI and/or PTSD | Mild | In some cases, substance use disorder, PTSD, major depressive disorder | Not reported | Not reported | Veterans |  | 27 veterans with mTBI and/or PTSD | 27 | Research team, clinicians, 'buddies' identified by patient | Mixed (PDAs and Mobile phones) | Community (Residential treatment facility and home) | Once daily, random times (Waking hours) | Async | No | Custom 32-item PROm derived from Symptom Checklist-6, the BriefCOPE, Beck Depression Inventory II and miscellaneous questions. Simple (1-5 Likert) check-in question over SMS post-discharge (1 = Great; 5 = Lousy) | The results of the pilot suggest that the mobile ecological momentary assessment and SMS messaging tools are feasible adjuncts to traditional mental treatment in the veteran population. Additional work is needed to establish the degree of clinical and economic value. PDA data retrieval often not timely enough to react to stress or crisis situations. Only 23% of PDA EMA data collection attempts were completed at random times; 89% for scheduled times. For SMS follow-up, 92%, 95% and 91% engagement at 30, 60 and 90 days; mean 72.2 days (SD 47.9) enrolled in follow-up SMS. |
| Bell, et al. | 2004 | Development of a telephone follow-up program for individuals following traumatic brain injury | Original - Descriptive | USA | To describe the development of a telephone follow-up program that addresses the needs of survivors of traumatic brain injury (TBI) and their families in the year following injury. | TBI | Moderate, Severe | Not explicitly reported | <1mo | 2 weeks post-discharge | Adults and families/carers (significant others) | Average age (SD) = 34.4y (13.6)  Sex (F) = 18%; 76% white | 84 | 84 | Research care manager | Telephony | Community | 7 planned contacts at 2 weeks, 4 weeks, 2 months, 3 months, 5 months, 7 months, 9 months | Sync | Yes - Use of proxy at each contact (family member or significant other) | Non-specific review of past and current concerns with triage/referral as required; structured interview addressing 17 domains: personal care, ambulation, travel, work, school, home management, leisure, social integration, cognitive and behavioral concerns, standard of living, financial independence, medical concerns, emotional function, alcohol use, drug use, legal issues, and spirituality | Median 4 contacts with patients and 4.5 with relatives over 9-month period Median call duration 34 min with TBI patients, 30 min with relatives. The study demonstrated that proactive, provider-initiated telephone follow-ups were feasible, and over 50% of participants required some level of intervention. The telephone follow-up program was found to be effective in bridging gaps in care, especially for those with geographic or financial barriers. The most frequent issues for participants included medical, work-related, cognitive-behavioral, financial, and emotional difficulties. The program was deemed feasible and beneficial, with participants and caregivers expressing appreciation for the support provided during the transition from hospital to community care. |
| Dombovy, et al. | 1997 | Recovery and rehabilitation following traumatic brain injury | Original - Descriptive | USA | To determine if functional, neuropsychological, and social outcome at 3 and 6 months in patients hospitalized following traumatic brain injury (TBI) could be ascertained via telephone follow-up, and assess use of rehabilitation services in this population. | TBI | Mild, Moderate, Severe | Undisclosed history of psychiatric diagnosis in 13.5% | 1-3mo | 2.5-3.5 months post-injury. | Adults | Mean 39.2y; 29.7%F; 85.1% white. | 74 | 74 | Nurse practitioner | Telephony | Community | 2 sessions, 15-30min in duration, at 3- and 6-months post-injury | Sync | No | Neurobehavioural Rating Scale (NRS) | Telephone deemed a cost-effective way to ascertain functional and neuropsychological outcomes in TBI survivors, and may identify those who may benefit from additional rehabilitation The study emphasized the importance of telephone follow-up to identify patients in need of further rehabilitation services and better manage long-term outcomes. |
